# Benchmarking medical laboratory performance on a global scale

**DOI:** 10.3389/fpubh.2024.1363957

**Published:** 2024-06-17

**Authors:** Wolfgang Huf, Mike Mohns, Eni Almeta, Rebecca Lister, Christoph Buchta, Svitlana Demyanets, Wolfgang Buchberger, Brigitte Ettl

**Affiliations:** ^1^Karl Landsteiner Institute for Clinical Risk Management, Vienna, Austria; ^2^Research Unit for Quality and Efficiency in Medicine, Institute for Public Health, Medical Decision Making and HTA, UMIT TIROL - University for Health Sciences and Technology, Hall in Tirol, Austria; ^3^Abbott GmbH, Wiesbaden, Germany; ^4^Austrian Association for Quality Assurance and Standardization of Medical and Diagnostic Tests (ÖQUASTA), Vienna, Austria; ^5^Department of Laboratory Medicine, Clinic Hietzing, Vienna, Austria; ^6^Department of Laboratory Medicine, Medical University of Vienna, Vienna, Austria

**Keywords:** benchmarks, diagnostic quality, diagnostic laboratory, clinical laboratory, patient safety, questionnaire, digitalization, automation

## Abstract

**Background and aims:**

Laboratory performance as a relative concept needs repetitive benchmarking for continuous improvement of laboratory procedures and medical processes. Benchmarking as such establishes reference levels as a basis for improvements efforts for healthcare institutions along the diagnosis cycle, with the patient at its center. But while this concept seems to be generally acknowledged in laboratory medicine, a lack of practical implementation hinders progress at a global level. The aim of this study was to examine the utility of a specific combination of indicators and survey-based data collection approach, and to establish a global benchmarking dataset of laboratory performance for decision makers in healthcare institutions.

**Methods:**

The survey consisted of 44 items relating to laboratory operations in general and three subscales identified in previous studies. A global sample of laboratories was approached by trained professionals. Results were analyzed with standard descriptive statistics and exploratory factor analysis. Dimensional reduction of specific items was performed using confirmatory factor analysis, resulting in individual laboratory scores for the three subscales of “Operational performance,” “Integrated clinical care performance,” and “Financial sustainability” for the high-level concept of laboratory performance.

**Results and conclusions:**

In total, 920 laboratories from 55 countries across the globe participated in the survey, of which 401 were government hospital laboratories, 296 private hospital laboratories, and 223 commercial laboratories. Relevant results include the need for digitalization and automation along the diagnosis cycle. Formal quality management systems (ISO 9001, ISO 15189 etc.) need to be adapted more broadly to increase patient safety. Monitoring of key performance indicators (KPIs) relating to healthcare performance was generally low (in the range of 10–30% of laboratories overall), and as a particularly salient result, only 19% of laboratories monitored KPIs relating to speeding up diagnosis and treatment. Altogether, this benchmark elucidates current practice and has the potential to guide improvement efforts and standardization in quality & safety for patients and employees alike as well as sustainability of healthcare systems around the globe.

## 1 Introduction

Laboratory performance is a relative concept, and therefore repetitive benchmarking is essential to continuously improve medical processes involving not only but also laboratory procedures. The basic idea of benchmarking is to establish a reference level, on which improvement efforts may build upon. But while this concept seems to be generally acknowledged, there appears to be a lack of practical implementation at a global level.

Several quality improvement initiatives have been launched over the years, some of which relate to general laboratory performance, others to specific aspects of laboratory performance. Among the former are the endeavor of the International Federation of Clinical Chemistry and Laboratory Medicine (IFCC) Working Group on Laboratory Errors and Patient Safety (WG-LEPS) and the Q-Probes program of the American College of Pathologists (1, 2). Among the latter are numerous external quality assessment (EQA) schemes (3). However, for reasons that are still not fully elucidated, none of these systems have globally been successful.

Plebani et al. have coined the term “quality indicator paradox” to describe the discrepancy between the general interest of laboratories to improve on efficiency, quality, and patient safety—and actual activity in this regard (4). The implementation of high-level concepts of laboratory performance in clinical practice seems to be hampered by a number of fundamental challenges that root in the complexities of the healthcare system. To install effective programs, a few key elements are needed but are typically not established (5): a clear vision and organizational alignment, appropriate skills for program management, resources to support the program, incentives to motivate participation, and a plan of action that articulates program objectives and metrics. Moreover, laboratory management methods historically have developed rather hands-on and only recently have been more widely addressed in the academic literature.

The number of key performance indicators (so called “quality indicators,” QIs) commonly used in laboratory medicine is rather limited (6, 7). Some exceptions are total numbers or proportions (e.g., number of patients, number of orders, number of samples, proportion of samples where the analysis was not possible because of errors in pre-examination processes), time measures that are relevant for clinical practice (e.g., various definitions of turn-around times, TATs), and resource measures relating to financial viability (e.g., number of full-time equivalents, laboratory space). Generally, the literature on medical laboratory performance benchmarking is still sparse (8).

To improve on this overall situation, the authors of this study started a multi-stage initiative to test the feasibility of a particular approach to global laboratory benchmarking. Its aim was to develop a survey-based approach for a comprehensive assessment of medical laboratory performance as a basis for measurably better health care. At the core of this initiative were a simple questionnaire and an active data elicitation approach with higher fidelity than simple online surveying. Since the global laboratory community exhibits quite some degree of heterogeneity, the implementation of data collection procedures was considered a specific strategic challenge.

Our general approach has already been described in previous publications (8, 9). Briefly, it has consisted of a general design phase and three iterative stages with adaptations in the sense of the Plan-Do-Check-Act (PDCA) cycle (10). In the general design phase, the constructs to be surveyed and the majority of items as well as the data collection process were defined with the help of focus groups. In the three implementation stages, both the survey and the data collection approach have been subject to review and adaptation.

In the first stage of implementation, the data collection approach was tested on a pilot sample, the results of which were published together with the questionnaire (8). In the second stage of implementation, the questionnaire itself was validated on a larger sample (9). This publication describes the third stage of implementation, where insights gained during stages one and two of implementation have led to the first global survey of this initiative.

**Figure 1:**
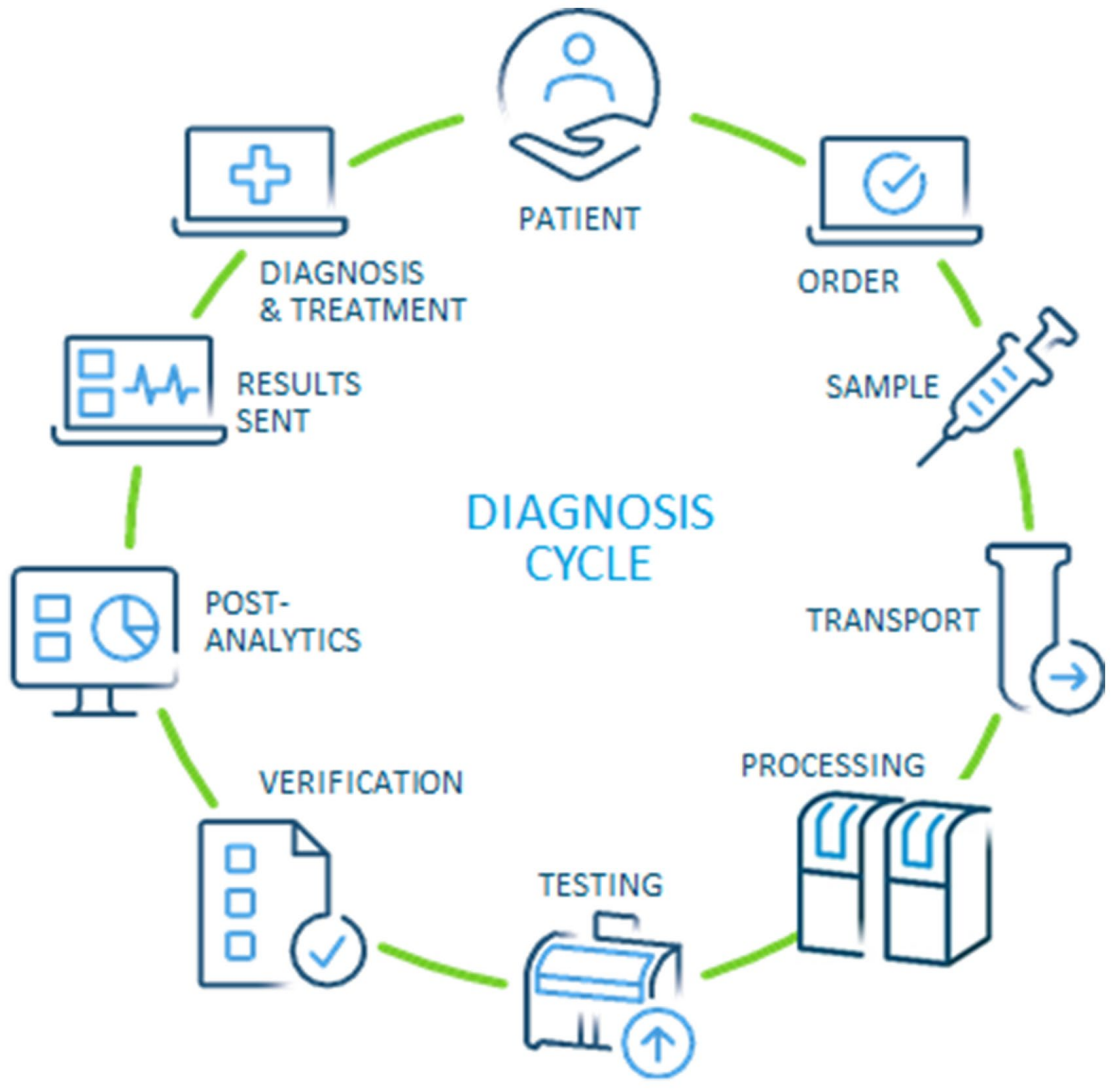
Modern diagnosis cycle, adapted from the original idea by Lundberg (11, 12).

Based on the original concept of Lundberg, our approach has aimed to capture all phases of a modern diagnosis cycle (11). Starting and ending with the patient (or healthy individual in case of health check), the modern version should include the steps of ordering, preparation of individuals, specimen collection, transportation, pre-examination processes in the laboratory, testing, technical verification, post-examination processes, and reporting of results to establish a diagnosis and treatment plan (Figure 1). Since the current stage of development of performance measures on a global scale is quite inconsistent, we designed the questionnaire to be as suitable as possible for all laboratories. Fortunately, the medical process *per se* is the same worldwide, which provides a common basis for interpretation.

## 2 Materials and methods

The questionnaire used for this study consisted of 44 items (see Supplementary material for item prompts). It was based on the questionnaire used for stages one and two, with some adaptations based on the feedback from those two stages aiming for maximum acceptance in the target audience (of laboratory professionals worldwide) while still allowing to measure the previously identified key dimensions of medical laboratory performance named “Operational performance,” “Integrated clinical care performance,” and “Financial sustainability” (8, 9). The iterative design process was based on previous knowledge from the literature, author experience, feedback from informal focus groups and respondents from stage one and two. The focus groups consisted of about 20 people each, varying slightly due to availability of participants, and included medical doctors, technicians, workflow experts, biologists, and laboratory directors.

A common theme appearing in all focus groups was diversity of terminology used among medical laboratory professionals. Optimally, medical laboratory professionals should use a common terminology consistent with the wording used in international certification/accreditation norms, e.g., ISO 15189, CLSI AUTO15, and CLSI AUTO17. As yet, however, this ideal state has not been reached and we felt the need to bridge this gap in order to elicit data globally as valid as possible taking into consideration the diversity of terminologies currently used in practice. The wording used in the questionnaire appeared to have the highest level of acceptance in the focus groups. Where necessary, we provide clarifications in the text below.

The target for this strategic effort were medical laboratories, the general approach aiming to be vendor independent. At stages one to three of this initiative, convenience samples were sought from laboratories with a broad range of diagnostic equipment providers, including Abbott, Roche, Siemens, Sysmex, Beckman, Werfen, Biomerieux, Becton Dickinson, Stago, and Diasorin. Specifically trained Abbott customer representatives were asked to approach general medical laboratories known to them both with and without Abbott equipment, requesting participation in the study. Given consent by the laboratory, the questionnaire was then filled out online using the platform SurveyMonkey with support of Abbott representatives. Only where it was not possible to complete the questionnaire directly online, the survey was completed on paper and then entered manually.

To increase the quality of the dataset used for subsequent analyses, a two-stage approach was chosen for data cleaning. As first stage, a correction loop was introduced which gave the representatives the opportunity to discuss the results with laboratory personnel and thus check the plausibility of data entered. The second stage consisted of univariate examination of the variables, in particular input variables for factor analysis, and the removal of highly implausible values [e.g., values of patients per full-time equivalent (FTE) ≥5,000].

Statistics and visualization were performed using the free software environment R version 4.1.2 (13). For descriptive statistics, the results are generally presented as numbers and percentages for nominal scale variables, median and inter-quartile range (IQR) for ordinal scale variables, and mean and standard deviation for numeric scale variables. In the results section, the focus is on subitems and aggregated items selected for confirmatory factor analysis, results beyond this can be found in the Supplementary material.

A set of 18 subitems was selected for dimensional reduction using exploratory factor analysis with OBLIMIN rotation. The resulting allocation of subitems and aggregated items to factors was then discussed and slightly adapted to conform with previous experience and model explainability for the target audience of laboratory professionals (9). In particular, due to feedback for some items we separated for aggregation individual subitems or sets of subitems from each other. We opted for this approach, in contrast to a solely data-driven approach, in order to increase acceptability and thus feasibility of the initiative as a whole. The resulting structure (see for selected subitems, aggregated items and primary factor loadings) was then submitted to confirmatory factor analysis using the R psych package (14).

## 3 Results

Overall, 920 medical laboratories from 55 countries across the globe responded to the survey (cf. Supplementary material, sections “Item 01—Location” and “Item 02—Laboratory type”). The top 12 countries accounted for roughly two thirds (67.6%, *n* = 622) of responses: South Africa (*n* = 65), India (*n* = 64), Saudi Arabia (*n* = 60), Indonesia (*n* = 58), Japan (*n* = 57), United Arab Emirates (*n* = 54), Thailand (*n* = 50), Vietnam (*n* = 50), Serbia (*n* = 47),Taiwan (*n* = 42), France (*n* = 41), and Greece (*n* = 34). About three quarters (76%, *n* = 697) were hospital laboratories (see Table 1), most of them from government hospitals (*n* = 401), about a third were private hospitals (*n* = 296), the rest were commercial laboratories (*n* = 223). Commercial laboratories were defined as medical laboratories “that are not associated with hospitals or healthcare facilities and that often provide a broad range of services over a wide geographical area” (15).

**Table 1:** Number of respondents.

Laboratory type	Frequency
Combined	920
Government hospital laboratory	401
Private hospital laboratory	296
Commercial laboratory	223

Laboratories served a mean of 1,670 patients per day (cf. Supplementary material, section “Item 02—Patients per day”). Government hospital laboratories and commercial laboratories were approximately the same size, with a mean of 2002 and 1910 patients per day, respectively. Private hospitals were on average half the size, with around 1,040 patients per day. Variability was high for all three types of laboratories (standard deviations around 2,000 to 3,000), due to strongly right skewed distributions.

Item 8 of the questionnaire examined the effects of COVID-19 on laboratory operations (cf. Supplementary material, section “Item 08—Effects of COVID-19”) inspired by NHS GIRFT Programme National Specialty Report Pathology (16). Roughly half of the laboratories established changes for the subitems probed: “Providing stronger guidance how to take samples correctly,” “Controlling quality in an appropriate and visible way,” “Labelling samples correctly so that the patient can be correctly identified,” “Delivering results within a clinically meaningful timeframe,” “Making results visible to all those who need to see them,” “Providing advice and support on interpretations and appropriate responses to results,” and “Ability of computer system and software to exchange and to make use of information across locations.” Almost two thirds of laboratories established changes for “Providing stronger guidance how to take samples correctly” (62.4%, *n* = 574). Changes were less pronounced for the other subitems, with “Ability of computer system and software to exchange and to make use of information across locations” being least pronounced (44.3%, *n* = 408).

Results for item 10 was concordant with the focus for improvement exhibited in the results for item 8. Almost all of the laboratories had a clear focus on “Sample quality” (93%, *n* = 860), “Sample volume” (91%, *n* = 835), “Labelling problems” (88%, *n* = 809), and “Use of inappropriate sample containers” (87%, *n* = 802). All other pre-laboratory indicators were less intensely monitored, with “Average of normal potassium vs. time from collection” ranking last (37%, *n* = 338).

### 3.1 Operational performance

**Figure 2:**
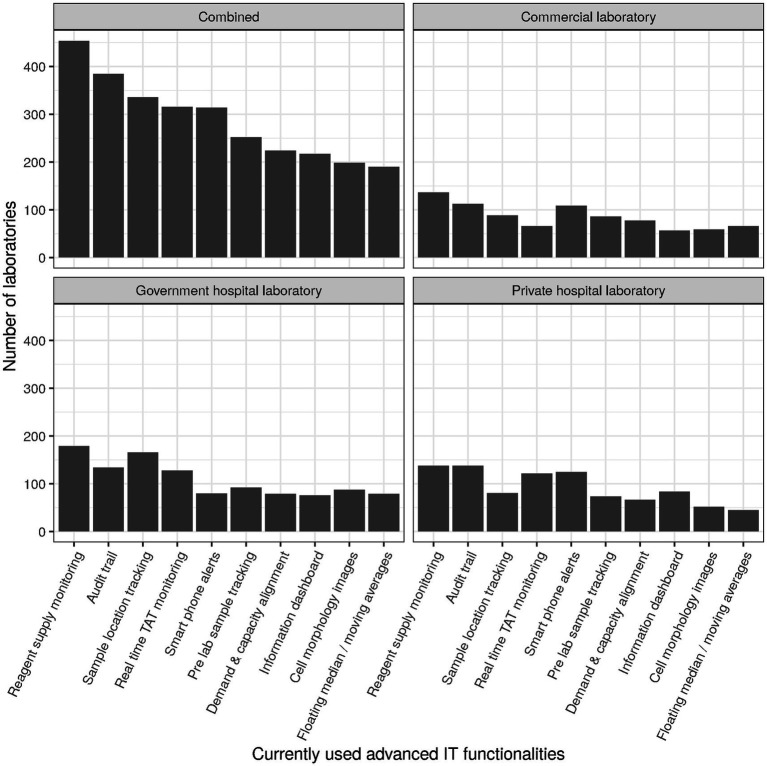
Numbers of laboratories currently using specific advanced IT functionalities.

**Figure 3:**
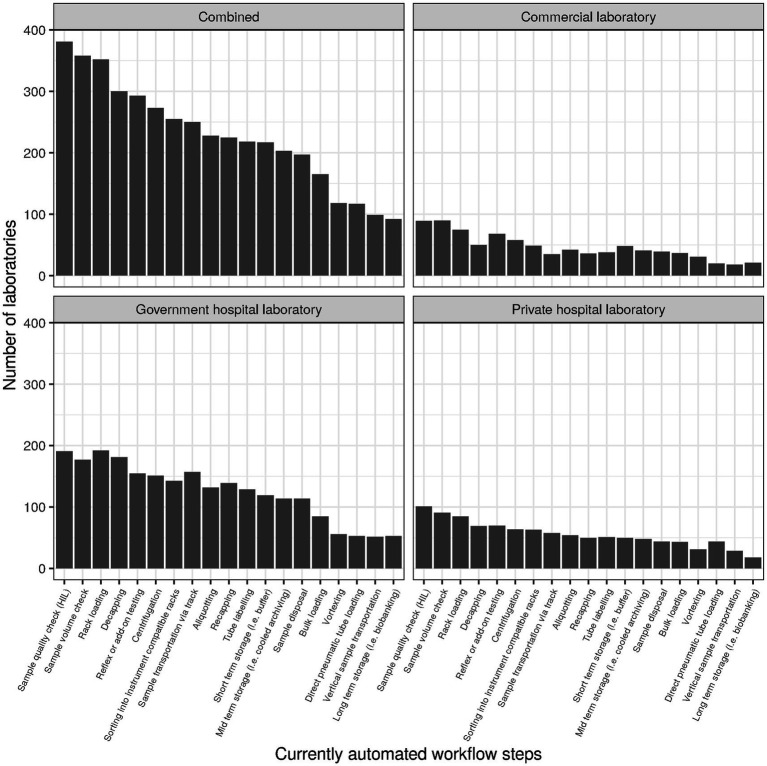
Number of laboratories currently using specific automated workflow steps.

Items subsumed under the header “Operational performance” during survey design roughly corresponded to items 10 through 22, with items for the subscale “Financial sustainability” being intermingled. For the subscale operational performance (cf. section Factor analysis), the following items were selected (cf. Table 2): patients per day (item 4) divided by full-time equivalents in clinical chemistry (item 42, subitem 1; referred to as “patients per FTE”), percentage of requests that are ordered electronically (item 15; “electronic ordering”), auto-verification rate for clinical chemistry (item 16, subitem 1; “auto-verification”), use of basic and advanced IT functionality (items 17 and 18, respectively; Figure 2), and degree of automation (item 19; Figure 3). For items 17 and 18, raw values before normalization could range between 0 (none currently in use) and 10 (all 10 subitems currently in use). Similarly, raw values could range between 0 and 19 for item 19.

**Table 2:** Questionnaire subitems and aggregated items loading on the three factors.

Operational performance	Integrated clinical care	Financial sustainability
4/42.1 Patients per FTE	16.2 Auto-validation	7.1–5 LEAN, Satisfaction
15 Electronic ordering	25 Services to physicians	7.6 Return on investment
16.1 Auto-verification	25.4 Diagnostic pathways	11 KPI utilization
17 Basic IT functions	25.9 Utilization guidance	11.2 Employee productivity
18 Advanced IT functions	27 Communication strategy	11.3 Workspace utilization
19 Degree of automation	28 Outreach strategy	23.2–4 Hospital utilization

**Figure 4:**
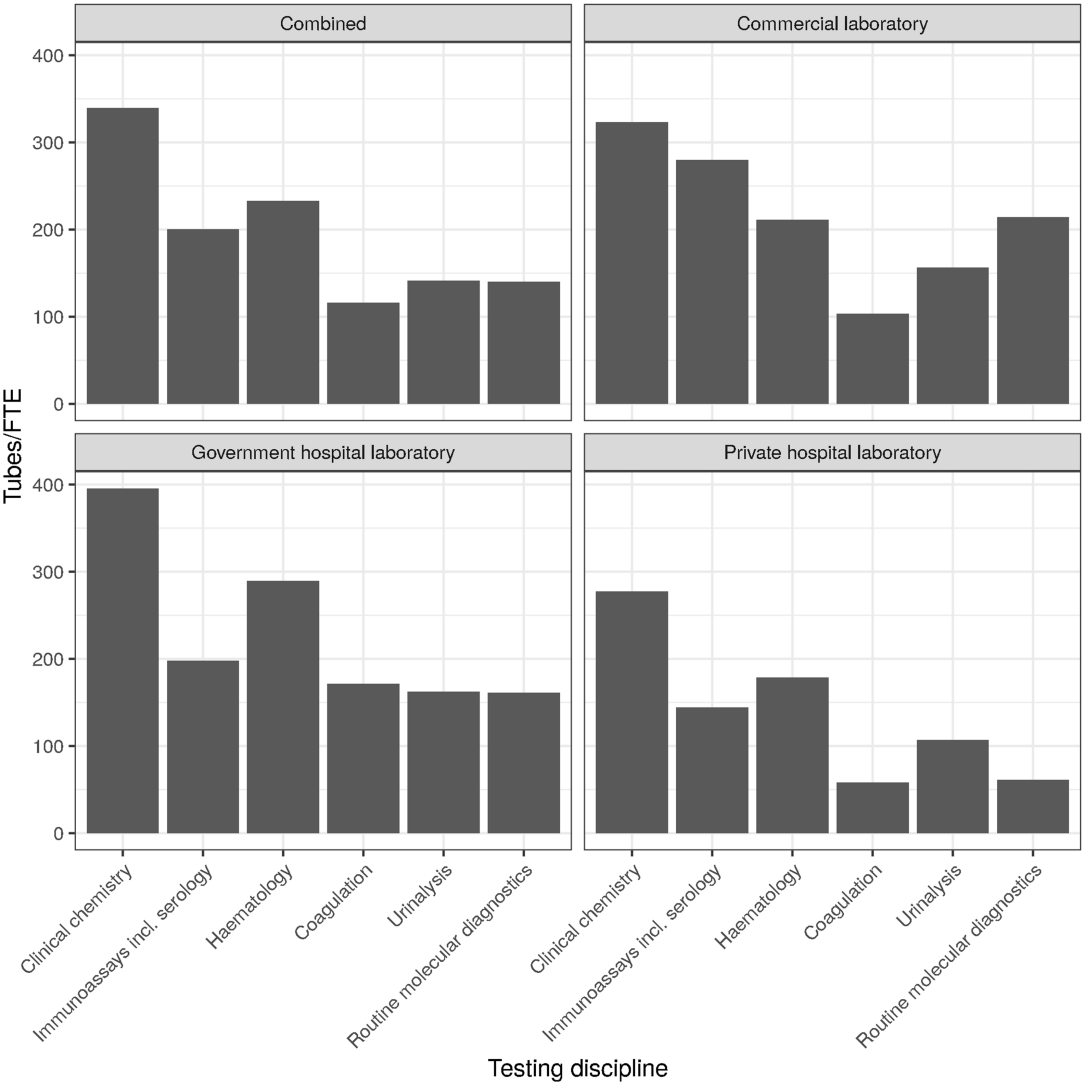
Employee productivity (tubes per FTE) by testing discipline.

The overall use of KPIs ranged between 20% and 89%, with turnaround-time (TAT) being at the top and work space utilization at the bottom. The top and bottom ranks were consistent overall and for the three laboratory types. Interestingly, employee productivity also ranked at the bottom, with only about a third of laboratories measuring it (cf. Figure 4 for an approximation of employee productivity by testing discipline). Commercial laboratories used it to a slightly higher degree (46%), but still much lower than TAT (87%), expired reagent stock (86%), or systems uptime/downtime (65%).

**Figure 5:**
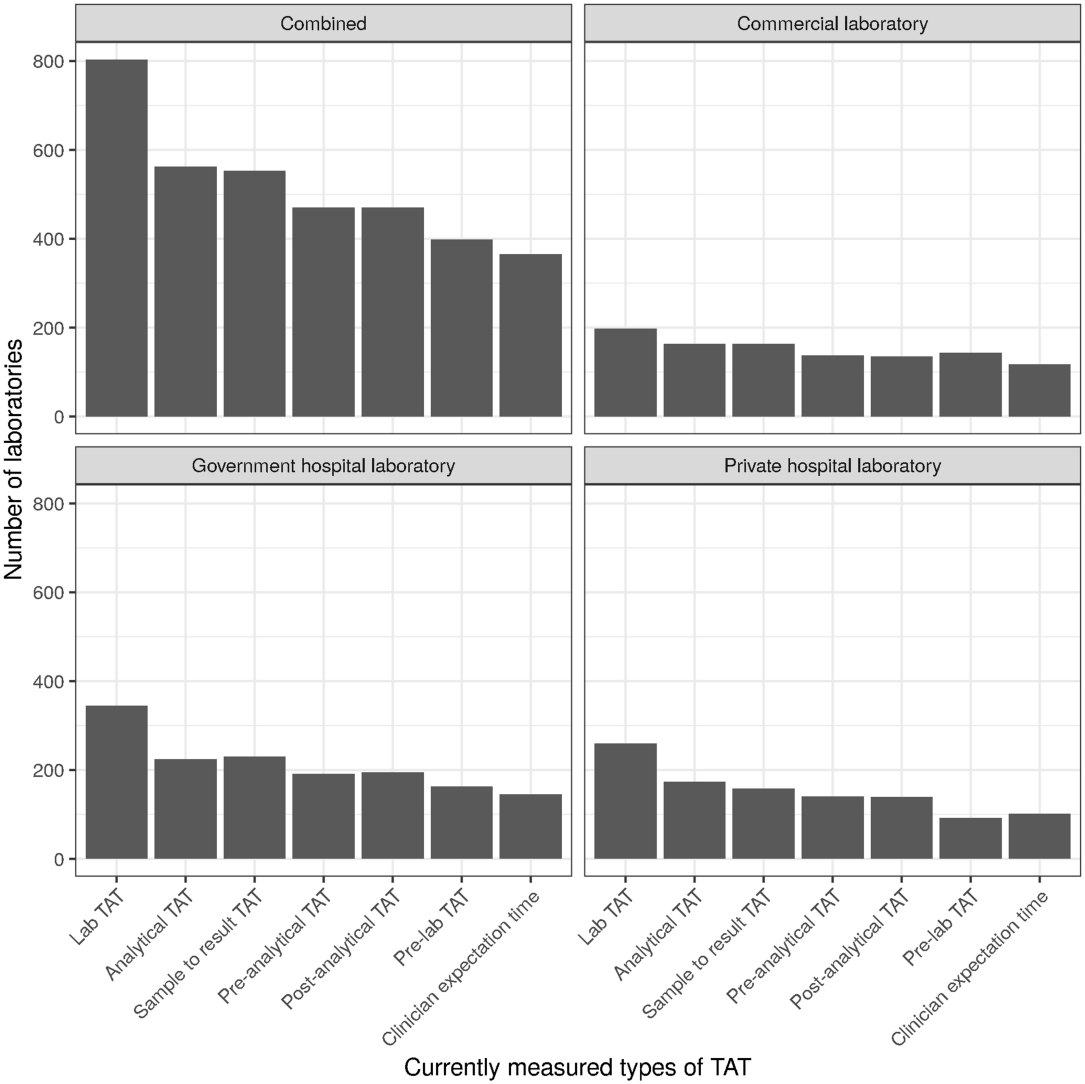
Numbers of laboratories currently measuring specific types of TAT.

For TAT being the top KPIs used by laboratories, the specific type of TAT used seemed to of interest. Most laboratories used “Lab TAT” (Figure 5), comprising the time between sample reception and result release [cf. Supplementary material, section “Item 12—Turn-around time (TAT)”]. Overall and largely consistent with the three laboratory types, “Clinician expectation time” (time from order generation to access of the results by the clinician/physician) was the TAT least monitored.

The values for patients per full-time equivalent (FTE; for clinical chemistry) had a mean of 452 and a standard deviation (SD) of 606, reflecting the right skewness of the laboratory size distribution (cf. Supplementary material, section “Factor analysis”). Performance was similar for government hospital laboratories (mean 483, SD 638), private hospital laboratories (mean 408, SD 561), and commercial laboratories (mean 458, SD 607). The median values were 267 for government hospital laboratories, and 200 for private hospital and commercial laboratories alike. IQR was about 400 for all three types of laboratories.

Electronic ordering was implemented on average for 57% of orders (SD 41%, median 70%, IQR 90%; cf. Supplementary material, section “Item 15—Electronic ordering”). For commercial laboratories the proportion was much lower (mean 37%, median 10%). Technical auto-verification rate on average was around 20–30% overall (SD 25–40%, median 0, IQR 50–70%) as well as for the three types of laboratories separately. As a note on terminology, we aimed to differentiate between IT supported review of “the results of examinations and evaluate them against IQC” and “available clinical information and previous examination results” with the terms “technical auto-verification” and “clinical auto-validation,” respectively.

Basic IT functions surveyed were used by about two thirds of laboratories, with the exception of “Tracking costs per tests” which was only used by about one third of laboratories (cf. Supplementary material, section “Item 17—Basic IT functionalities”). Most frequently, laboratories used “Age/gender related rules” (80%, *n* = 740), “Turnaround time monitoring” (80%, *n* = 733), and “QC Monitoring” (79%, *n* = 724). In contrast, advanced IT functions overall were used by only about one third of laboratories or less in a relatively consistent manner (cf. Supplementary material, section “Item 18—Advanced IT functionalities,” and Figure 2). Only “Reagent supply monitoring/ordering” (49%, *n* = 454) and “Audit trail (end to end traceability for reagents, controls and consumables)” (42%, *n* = 385) were used by close to half of laboratories. The function least used was “Patient result monitoring using floating median/moving averages” (21%, *n* = 190). Regarding terminology, “floating median” is not described in ISO 15189 but implemented in middleware solutions of various vendors.

Of the 19 subitems surveyed for “Item 19-Automation,” most were used by only one third of laboratories or less, with the notable exceptions of “Sample quality check (HIL)” (41%, *n* = 381), “Sample Volume check” (39%, *n* = 358), and “Rack loading” (38%, *n* = 352). The subitem least considered was “Long term storage (i.e., Biobanking)” (10%, *n* = 92). Degree of automation was similar overall and for the three types of laboratories (cf. Supplementary material, section “Item 19—Automation”).

### 3.2 Integrated clinical care performance

**Figure 6:**
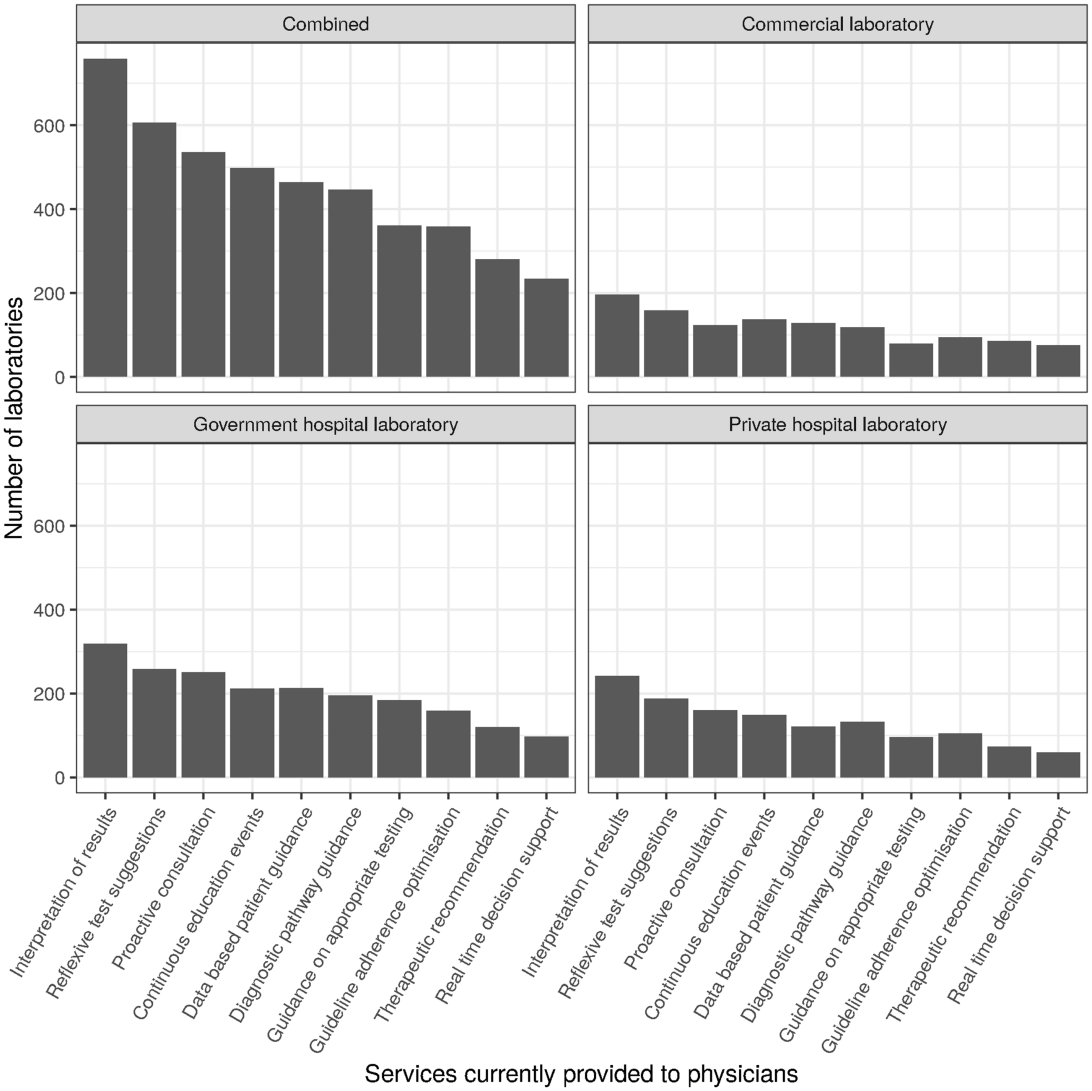
Numbers of laboratories currently providing specific services to physicians.

Items subsumed under the header “Integrated clinical care performance” during survey design roughly corresponded to items 23 through 28. For the corresponding factor analysis subscale (cf. section Factor analysis), the following items were selected (cf. Table 2): clinical auto-validation for clinical chemistry (item 16, subitem 2; “auto-validation”), services provided to physicians (item 25; Figure 6), communication strategy (item 27) and outreach strategy (item 28). For services provided to physicians (item 25), we separately examined services that can typically be established unilaterally by the laboratory (“services to physicians”; subitems 1–3, 5–8, and 10; raw values ranging between 0 and 8) and services that typically need more extensive collaboration and consensus with clinicians in order to gain acceptance. For the latter we differentiated between those mostly examining sets of parameters (diagnostic pathways; subitem 4) and those mostly examining individual parameters (utilization guidance; subitem 9).

Clinical auto-validation rate was around 10% overall (SD around 25%). For the three types of laboratories mean auto-validation rates were at about 10, 5, and 15% for government hospital laboratories, private hospital laboratories, and commercial laboratories, respectively. Median was 0 overall and for all three types of laboratories, with interquartile ranges also tending to be 0 due to the low percentage of clinical auto-validation in the entire field.

Services provided to physicians sampled in the survey were “Interpretation of results,” “Reflexive test suggestions,” “Proactive consultation on complex patient cases,” “Diagnostic pathway guidance,” “Optimization of adherence to diagnostic guidelines,” “Therapeutic recommendations,” “Real time decision support using clinical algorithms,” “Guidance based on historical patient results,” “Guidance on over- and under-utilization of testing,” and “Continuous education events.” They were provided in roughly one quarter to two thirds of cases, with “Interpretation of results” being a notable outlier at the top (82%, *n* = 759) and “Real time decision support using clinical algorithms” at the bottom (25%, *n* = 234). Top and bottom places were the same for all types of laboratories (Figure 6).

Subitem 4 of item 25 (“Diagnostic pathway guidance”) related to guiding clinicians via collaboration and consensus regarding specific sets of parameters to be used for optimizing the diagnostic part of the diagnosis cycle. About half of laboratories surveyed supported clinicians/physicians in this way. The provision of diagnostic pathway guidance was essentially independent of laboratory type.

Subitem 9 of item 25 (“Guidance on over- and under-utilization of testing”) related to guiding clinicians/physicians via collaboration and consensus regarding the use of individual parameters for clinical and economic purposes. Less than half of laboratories supported clinicians/physicians and the hospital in this way. Service provision was highest in government hospital laboratories (46%, *n* = 185) and about 10% lower in private hospital laboratories (33%, *n* = 97) and commercial laboratories (35%, *n* = 79).

Communication approaches sampled in the survey were “Email,” “Social media,” “Website,” “Newsletter,” “Print media,” and “Events” (cf. Supplementary material, section “Item 27—Communication channels”). Over all approaches, uptake was relatively high as compared to other items sampled in the survey. Digital approaches as well as events were used by about half of laboratories or more (46–71%). In this case, digitalization appears to be well established, with print media being most seldomly used (44%) as tools of communication. Notably, social media was lacking in government hospitals (26%, *n* = 104) vs. private hospitals (61%, *n* = 182) and commercial laboratories (74%, *n* = 165).

Approaches used for outreach regarding public health topics were “Disease surveillance and/or outbreak management,” “Partnership with public or private payers to leverage lab data for risk management,” “Outcome based pricing or reimbursement schemes that reward labs for value creation,” “Preventative health and wellness programs,” “Contribution to Population Health Initiatives,” and “Referral of patients to others services or specialists” (cf. Supplementary material, section “Item 28—Outreach strategy”). These were consistently used in less than half of laboratories, with “Disease surveillance and/or outbreak management” exhibiting the highest uptake (45%, *n* = 412) and “Outcome based pricing or reimbursement schemes that reward labs for value creation” the lowest (13%, *n* = 119). Particularly interesting in the context of the pandemic, the use of “Disease surveillance and/or outbreak management” was lower in commercial laboratories (35%, *n* = 79) than government (45%, *n* = 181) or private hospital laboratories (51%, *n* = 152).

### 3.3 Financial sustainability

Items subsumed under the header “Financial sustainability” were interspersed through the entire survey and aimed in a wider sense at identifying sustainability of current management practice and future preparedness of the laboratory. For the corresponding factor analysis subscale (cf. section Factor analysis), the following items were selected (Table 2): item 7, subitems 1 through 5, representing the general idea of satisfaction; item 7, subitem 6, identifying measurement of return on investment; item 11, subitem 2, identifying measurement of employee productivity; item 11, subitem 3, identifying measurement of work space utilization; item 11, subitems 1 and 3 through 10, identifying use of KPIs in general; and item 23, subitems 2 through 4, identifying measurement of hospital utilization (via delays for inpatient procedures and readmissions).

The general idea of satisfaction (via LEAN, surveys, and training) was represented by about two thirds of laboratories, largely independent of laboratory type. Only LEAN was represented to a much lower degree, at about one quarter of laboratories, also independent of laboratory type. “Continuous training/development program for employees” was at the top in all laboratory types with at least 80% (overall 84%, *n* = 777) of laboratories using it.

“Return on investment (e.g., TVO, TCO)” was used to a relatively low degree, by less than one fifth of laboratories (18%, *n* = 168) overall. Its use was lowest in government hospital laboratories (14%, *n* = 58), followed by private hospital laboratories (18%, *n* = 54). A quarter of commercial laboratories (25%, *n* = 56) used calculations regarding return on investment as best practice tools.

The overall use of KPIs ranged between 20% and 89%, with turnaround-time (TAT) being at the top and work space utilization at the bottom. The top and bottom ranks were consistent overall and for the three laboratory types. Interestingly, employee productivity also ranked at the bottom, with only about a third of laboratories measuring it (Figure 4 for an approximation by testing discipline). Commercial laboratories monitor employee productivity to a slightly higher degree (46%, *n* = 103), but still much lower than TAT (87%, *n* = 195), expired reagent stock (86%, *n* = 191), or systems uptime/downtime (65%, *n* = 145).

For TAT being the top KPIs used by laboratories, the specific type of TAT used was of interest. Most laboratories used “Lab TAT” (Figure 5), comprising the time between sample reception and result release [cf. Supplementary material, section “Item 12—Turn-around time (TAT)”]. Overall and largely consistent with the three laboratory types, “Clinician expectation time” (time from order generation to access of the results by the clinician/physician) was the TAT least monitored.

Regarding healthcare system KPIs (cf. Supplementary material, section “Item 23—Influence on healthcare system”), uptake was generally low (about 10 to 30% overall). For factor analysis we focused on hospital resource utilization parameters (subitems 2 through 4): “Delays of Surgical Procedures,” “Discharge delays; Ward Length of Stay,” and “Reducing readmissions.” These were highest in private hospital laboratories (17–23%) and lowest in commercial laboratories (3–8%) with government hospital laboratories in between (12–17%). Concordantly, “Speeding up diagnosis and treatment” was in the focus of only 15–22% of laboratories.

### 3.4 Factor analysis

In line with our previous benchmarking studies, exploratory factor analysis turned out to be a relevant basis for confirmatory factor analysis, the others being previous knowledge and feedback from respondents. For confirmatory factor analysis (CFA) a balanced set of input variables tended to achieve maximum acceptance. Input variables were thus either subitems (as numerical variables or Boolean variables converted to numerical) or sums of subitems, all input variables for factor analysis being independently normalized.

The resulting subitems and aggregated items are listed in Table 2. The factor “Operational performance” roughly relates to laboratory operations, the factor “Integrated clinical care” corresponds to interaction with medical personnel outside the laboratory, and the factor “Financial sustainability” practically stands for resilience or sustainability of laboratory operations. While not stellar, fit indices were in acceptable ranges (Table 3).

**Table 3:** Fit measures of confirmatory factor analysis.

Fit measure	Value
RMSEA	0.06
CFI	0.91
SRMR	0.08

**Figure 7:**
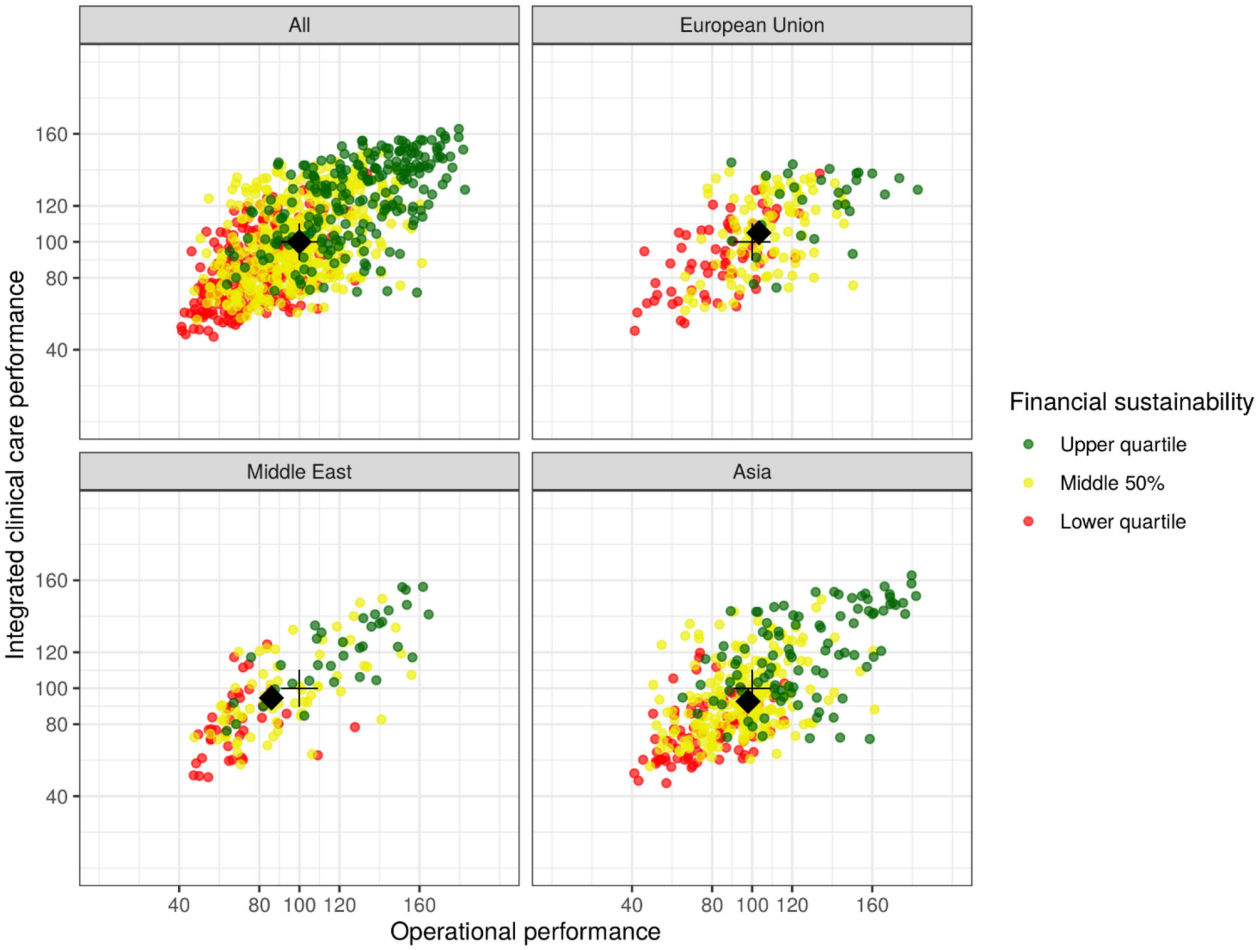
Scatterplot of subscale values representing three key dimensions of medical laboratory performance for all observations and three subgroups (European Union, Middle East, Asia), with each point representing one individual laboratory on the three subscales; its approximate positioning in relation to others is identifiable by value or color for the lower quartile (<80 or red), middle 50% (80–120 or amber), or upper quartile (>120 or green). The black crosses identify subscale medians for the overall dataset, while the black diamonds identify subgroup medians.

Projections to all three subscales can be found in Figure 7 (a normally distributed scatter with an SD of 2.5 added for visualization), with the medians for the subscales set to 100 and the spread adjusted so that about half of observations for each subscale are in the range of 80 to 120. Correlations were 0.67 between subscales “Operational performance” and “Integrated clinical care performance,” 0.71 between subscales “Operational performance” and “Financial sustainability,” and 0.62 between subscales “Integrated clinical care performance” and “Financial sustainability.” To account for model explainability in the target audience (cf. section Materials and methods), correlations are slightly higher than expected (Table 4).

**Table 4:** Correlation of subscales.

	OP	ICCP	FS
OP	1.00	0.68	0.71
ICCP	0.68	1.00	0.62
FS	0.71	0.62	1.00

To give a very rough idea of global variation, Figure 7 not only shows the entire dataset (“All”) but also three subgroups that exhibit reasonably high numbers observations (“European Union,” “Middle East,” “Asia”). The black crosses in all four subfigures identify subscale medians for the overall dataset, while the black diamonds identify subgroup medians. For the subgroup “European Union” (*n* = 191), the medians of the subscales “Operational performance” and “Integrated clinical care performance” are slightly higher than the corresponding global medians. For the subgroups “Middle East” (*n* = 142) and “Asia” (*n* = 379) the medians are slightly lower than the global medians for both subscales.

## 4 Discussion

Transversal and longitudinal comparison routinely aids in the interpretation of individual patients results, but both are not commonly used for individual laboratory assessment. The core of the problem does not appear to be a lack of theoretic understanding in the laboratory community, but rather the lack of execution (4). This results in the general lack of benchmarking data, with which to compare various features of one’s own laboratory operations—and the initial impetus for our stepwise approach culminating in this publication.

Over the course of the four years of this study, the authors have directly experienced both the facilitating and inhibiting factors with regard to global laboratory benchmarking. Among the former certainly are the professional ethics of the medical laboratory community. The latter include, above all, the considerable heterogeneity of the field, making it very difficult to identify a commonly accepted set of benchmarking parameters in combination with a sustainable data collection process. Overall, it can be stated that there is reason for hope, but also a long road ahead.

The results of this study, as of its precursor studies (8, 9), can be viewed from two perspectives: firstly, 920 laboratories actively shared performance data, which are described extensively in the Supplementary material; secondly, the available data allowed some validation of the general process and procedures used for data collection. In the authors’ view, the last aspect is probably more important because of the necessity to establish a robust and sustainable process for long-term longitudinal observation. But even that might not be enough, and legal obligation might be necessary, as is the case with external quality assessment (EQA) programs in some countries (17).

Following the pilot study for the region of Germany, Austria, and Switzerland, the follow-up study for Europe, the Middle East, and Africa, and this survey on a global level, resources for the first global repeat appear not to be available right now. But only a global estimate can truly benchmark the laboratory community as a whole, and the next step would be an attempt towards representativity of the sample—at least in some countries to begin with. Furthermore, while internal validity of the questionnaire subscales “Operational performance,” “Integrated clinical care performance,” and “Financial sustainability” have been established at this point, future studies should examine external validity and robustness against adaptations of the questionnaire. The latter are deemed relevant to stay on top of the demands of the ever-changing world of laboratory medicine.

As in the past, quality is likely to remain the main focus of medical laboratories, while speed has traditionally been the need of referring clinicians (18). This discrepancy may have been reduced to some extent during the recent pandemic but will still be an area for improvement. The need to involve the laboratory in the entire diagnosis cycle (Figure 1) appears to be a crucial requirement for improving, among others, performance, quality & safety for patients and professionals alike, and sustainability in the entire healthcare system (11, 12). In the 1980s, Lundberg described the brain-to-brain loop consisting of the steps of ordering, sample collection, identification, transportation, preparation, analysis, reporting, interpretation, and action. The main difference to a modern view (Figure 1) is that the opportunities created by digitalization and automation can be used to effectively increase quality and patient safety—and reduce cost for the healthcare system.

**Figure 8:**
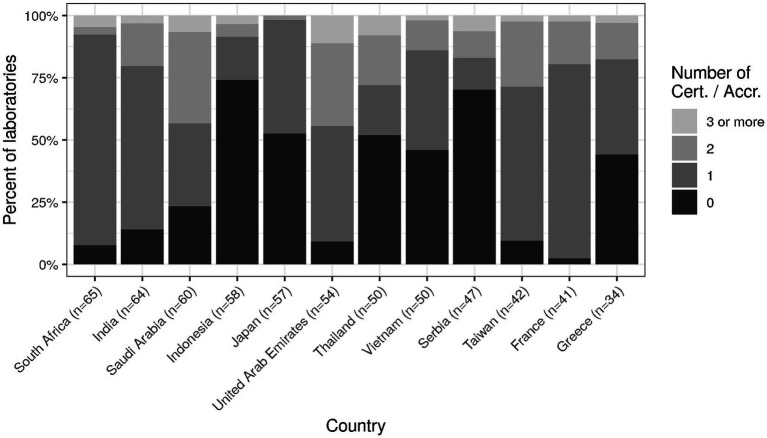
Holding of (multiple) international certification/accreditation for top twelve countries.

Some concrete measures can be taken regarding this context. First, the international trend towards certification (e.g., ISO 9001) or accreditation (e.g., ISO 15189) should be strengthened (19, 20). Currently, the level is still rather low with more than half of laboratories in some countries holding no international certification/accreditation at all (Figure 8). The efficiency of laboratory performance should not only take into account costs but also time expenditure (e.g., in the form of turn-around times) in order to balance this with high quality output (21). Both requirements are easier to meet with a high degree of digitalization and automation, which currently still have room for improvement (cf. Figures 2, 3). Indeed, the median for both clinical auto-validation and technical auto-verification is 0 for all testing disciplines (cf. Supplementary material, section “Item 16—Verification/Validation”), i.e., more than half of all results are neither auto-validated nor even auto-verified. Since humans are not very good at repetitive tasks, but machines are, this is an interesting finding in the 21st century.

Laboratory medicine as a field seems surprisingly slow to catch on to digitalization as a macro trend. On the one hand, there exist fully digitalized mega-laboratories, on the other hand, there is a relatively low degree of digitalization overall. This becomes evident in the survey, e.g., in items 8, 17, and 18 (cf. corresponding sections in Supplementary material). According to the responses to item 8, effects of COVID-19 lead to improvements in preanalytical quality (62%) but to a rather low degree to improvements in computer systems (44%). At least one fifth of laboratories did not even have basic IT functionality consistently implemented (item 17, range of implementation 35–80%), and advanced IT functionality was implemented by a maximum of 49% of laboratories (item 18, Figure 2).

With factor analysis and the three subscales of “Operational performance,” “Integrated clinical care performance,” and “Financial sustainability,” we aimed to give laboratory leadership an orientation about how to communicate value generation strategies. As can be expected, effects by changes in internal processes (“Operational performance”) correlate with changes to processes with external personnel (“Integrated clinical care performance”), which correlate with future preparedness of the laboratory as a whole (“Financial sustainability”). Improving on quality for the patient thus goes hand in hand with economic considerations.

As far as economic considerations are concerned, the future field of activity for the laboratories appears to lie at least to a considerable extent outside of the laboratory itself. The direct cost of the laboratory typically amounts to only about 2 % of healthcare costs (22, 23). Considering on the other hand that laboratory results influence clinical decisions to a large degree, and clinicians/physicians have a considerable potential to influence specific costs, the quality of upstream and downstream decisions seems to be an aspect that has received too little attention so far (24, 25). Surprisingly, only about two thirds of laboratories provide services to physicians beyond communication of test results and some interpretation in a consistent manner (cf. Supplementary material, section “Item 25—Services to physicians”). Concrete steps in this direction would add direct value and include more guidance on diagnostic pathways, optimization of adherence to diagnostic guidelines, therapeutic recommendations, and real time decision support using clinical algorithms.

Apart from the advantage of improving patient safety, including tools like clinical decision support has the potential to immediately reduce healthcare costs (26). On a longer timeframe, one might use systems to specifically target human attention to increase safety in high-throughput environments (27, 28). Interestingly, there is some evidence that combinations of human and computer intelligence leads to better results than either of the approaches alone (29). This might also be of interest when examining ways to reduce length of stay, delays or readmissions in hospitals, and in particular speeding up diagnosis and treatment. However, less than a quarter of laboratories tend to focus on these upstream and downstream opportunities to create value for institutions and patients alike (cf. Supplementary material, section “Item 23—Influence on healthcare system”).

The main limitations of this study relate to the process of data generation and the less than ideal discriminatory power of item formulations (30). However, both are at least to some degree due to practicability and thus represent a compromise. Results nevertheless should be interpreted with appropriate caution, particularly for values that are inherently difficult to determine (e.g., regarding personnel distribution over testing disciplines).

A truly representative sample of laboratories on a global scale would require an even higher number of laboratories and completely independent interviewers. The training effort needed and the costs would be immense. Regarding the discriminatory power of item formulations, we had to balance the specificity of the items against the comprehensibility in a host of different cultures in different parts of the world. In psychometrics, localization of questionnaires (i.e., their adaptation to a specific cultural, linguistic or otherwise regional context) on the one hand is an important aspect. Extensive localization on the other hand slows down the roll-out of questionnaires and requires way more resources than available for benchmarking efforts today.

Typical challenges for questionnaire studies include various forms of bias (e.g., social desirability bias—respondents answer as they think they should), low reliability, and low construct fit. All of these challenges adversely affect measurement quality. In this study, we aimed to reduce variability and increase relevance for respondents by training the interviewers and adapting the questionnaire to the current needs of the field. The latter related to the corona virus pandemic and aimed to increase comprehensibility by combining relevant subitems into items. On the other hand, this approach increases the correlation between neighboring subitems and thus may reduce the quality of the data collected.

With respect to the specific wording of the questionnaire, the discussion has gone on. To elicit data as valid as possible while simultaneously keeping the motivation for survey completion high, we opted for a terminology used in colloquial language of the medical laboratory community. This strength in turn introduces some obscurity of terminology. We feel that the community as a whole should work towards a clear common language, as, e.g., used by ISO 15189. The journey towards this goal, however, appears to be a long one in the face of the still relatively low international uptake of international accreditation.

Correspondingly, great caution is warranted when comparing results of medical laboratory subgroups. Apart from differences regarding legal requirements, cultural differences play a key role in adequate calibration and interpretation of questionnaire results. Taking as an example the above mentioned social desirability bias, one might ponder the need to validate the questionnaire for various cultural subgroups. One the one hand, this would probably give more accurate results, while on the other hand possibly rising cost to a level that compromises economic viability of academic studies of this kind. Practically, due to the questionnaire design process using diverse focus groups we expect global bias to be relatively low (in relation to the resources available for the entire modelling process) but variance in the combined dataset to be higher than in a theoretically ideal setting.

All in all, we balanced the various influences in the design of the questionnaire and data collection as well as possible and hope that this study will help to lay the foundation for future benchmarking studies in laboratory medicine. The main lesson learned from this study is that establishing and maintaining laboratory benchmarking on a global level is an extremely challenging task. The options are essentially to increase coverage by reducing data quality (e.g., by using an online-only format) or to increase data quality (and cost) by using some form of human-to-human interaction. Focusing on data quality as the priority, we mainly opted for the latter—but right now do not see an immediate way forward for this approach due to its high need for human resources. It remains to be seen whether future initiatives can find an even better balance between data quality, coverage, and cost.

Overall, it seems clear that laboratory benchmarking is needed to advance the field of laboratory medicine as a whole. Although quality has historically been in the focus of laboratory operations, the relatively low uptake of international certification/accreditation (such as ISO 9001 and ISO 15189) as well as EQA schemes (cf. Supplementary material, sections “Item 05—Certification / Accreditation” and “Item 06—External Quality Assessment”) testify to this need. Standardized criteria and indicators for structure, process and result quality combined with efficient data collection processes could accelerate the further development of laboratory medicine and thus effectively support sustainability of healthcare systems around the globe.

## Data availability statement

The datasets presented in this article are not readily available because of the need for anonymity of individual laboratories. Requests to access the datasets should be directed to mike.mohns@abbott.com.

## Author contributions

WH: Conceptualization, Formal analysis, Writing – original draft. MM: Conceptualization, Project administration, Writing – original draft. EA: Project administration, Writing – review & editing. RL: Writing – review & editing. CB: Writing – review & editing. SD: Writing – review & editing. WB: Writing – review & editing. BE: Writing – review & editing.
