# Supplementary material for: Benchmarking medical laboratory performance on a global scale
Source: Front Public Health. 2024 Jun 17;12:1363957. doi: 10.3389/fpubh.2024.1363957 (PMC11215183; doi:10.3389/fpubh.2024.1363957)
Supplement: Supplementary file 1 [file Data_Sheet_1.ZIP › Supplement_2023-1231-5484.html]

Benchmarking medical laboratory performance on a global scale


# Benchmarking medical laboratory performance on a global scale

#### Supplementary Material

#### Document version 2023-1231-5484

# Introduction

This document contains descriptive statistics by survey item and combined statistics pertaining to factor analysis. To keep respondents anonymous, some items (in particular free text items) were not analyzed for this study. This report was created using R version 4.1.2, R Markdown package version 2.11, and RStudio version 1.4.1106 on Linux Ubuntu 16.04.7 LTS. It is best viewed with Chrome based browsers (e.g. Chrome, Chromium, Edge).

# Items 01-10

## Item 01 - Location

### Prompt

Your location (pick one):

- …

### Results

| Answer | Frequency |
| --- | --- |
| South Africa | 65 |
| India | 64 |
| Saudi Arabia | 60 |
| Indonesia | 58 |
| Japan | 57 |
| United Arab Emirates | 54 |
| Thailand | 50 |
| Vietnam | 50 |
| Serbia | 47 |
| Taiwan | 42 |
| France | 41 |
| Greece | 34 |
| Malaysia | 31 |
| Iraq | 24 |
| Italy | 23 |
| Spain | 22 |
| Romania | 20 |
| Pakistan | 14 |
| Czechia | 13 |
| South Korea | 13 |
| Russia | 12 |
| Portugal | 10 |
| United Kingdom | 10 |
| Chile | 9 |
| Hong Kong | 9 |
| Netherlands | 9 |
| Bulgaria | 8 |
| El Salvador | 8 |
| Slovakia | 6 |
| Austria | 4 |
| Namibia | 4 |
| Qatar | 4 |
| Uruguay | 4 |
| Australia | 3 |
| Finland | 3 |
| Montenegro | 3 |
| Nigeria | 3 |
| Philippines | 3 |
| Germany | 2 |
| Kenya | 2 |
| New Zealand | 2 |
| Norway | 2 |
| Poland | 2 |
| Sweden | 2 |
| Switzerland | 2 |
| Turkey | 2 |
| Uganda | 2 |
| Albania | 1 |
| Bangladesh | 1 |
| Grenada | 1 |
| Ireland | 1 |
| Latvia | 1 |
| Lithuania | 1 |
| North Macedonia | 1 |
| Rwanda | 1 |

## Item 02 - Laboratory type

### Prompt

Laboratory classification (pick one):

- Government Hospital Laboratory
- Private Hospital Laboratory
- Private Commercial Laboratory

### Results

| Type | Frequency |
| --- | --- |
| Combined | 920 |
| Government Hospital Laboratory | 401 |
| Private Hospital Laboratory | 296 |
| Commercial Laboratory | 223 |

#### Laboratory type by location

|  | Government Hospital Laboratory | Private Hospital Laboratory | Commercial Laboratory |
| --- | --- | --- | --- |
| South Africa | 18 | 6 | 41 |
| India | 2 | 33 | 29 |
| Saudi Arabia | 43 | 16 | 1 |
| Indonesia | 11 | 44 | 3 |
| Japan | 31 | 26 | 0 |
| United Arab Emirates | 8 | 29 | 17 |
| Thailand | 25 | 22 | 3 |
| Vietnam | 27 | 18 | 5 |
| Serbia | 39 | 5 | 3 |
| Taiwan | 10 | 25 | 7 |
| France | 15 | 1 | 25 |
| Greece | 24 | 4 | 6 |
| Malaysia | 5 | 25 | 1 |
| Iraq | 3 | 2 | 19 |
| Italy | 15 | 2 | 6 |
| Spain | 22 | 0 | 0 |
| Romania | 11 | 0 | 9 |
| Pakistan | 6 | 4 | 4 |
| Czechia | 7 | 1 | 5 |
| South Korea | 7 | 3 | 3 |
| Russia | 7 | 2 | 3 |
| Portugal | 9 | 1 | 0 |
| United Kingdom | 10 | 0 | 0 |
| Chile | 6 | 2 | 1 |
| Hong Kong | 1 | 1 | 7 |
| Netherlands | 5 | 3 | 1 |
| Bulgaria | 4 | 0 | 4 |
| El Salvador | 0 | 5 | 3 |
| Slovakia | 2 | 0 | 4 |
| Austria | 1 | 1 | 2 |
| Namibia | 0 | 3 | 1 |
| Qatar | 1 | 1 | 2 |
| Uruguay | 2 | 2 | 0 |
| Australia | 3 | 0 | 0 |
| Finland | 2 | 1 | 0 |
| Montenegro | 3 | 0 | 0 |
| Nigeria | 0 | 1 | 2 |
| Philippines | 1 | 1 | 1 |
| Germany | 1 | 1 | 0 |
| Kenya | 1 | 1 | 0 |
| New Zealand | 2 | 0 | 0 |
| Norway | 2 | 0 | 0 |
| Poland | 1 | 0 | 1 |
| Sweden | 2 | 0 | 0 |
| Switzerland | 1 | 0 | 1 |
| Turkey | 1 | 1 | 0 |
| Uganda | 0 | 1 | 1 |
| Albania | 0 | 1 | 0 |
| Bangladesh | 0 | 0 | 1 |
| Grenada | 0 | 0 | 1 |
| Ireland | 0 | 1 | 0 |
| Latvia | 1 | 0 | 0 |
| Lithuania | 1 | 0 | 0 |
| North Macedonia | 1 | 0 | 0 |
| Rwanda | 1 | 0 | 0 |

## Item 03 - Part of chain or network

### Prompt

Is your laboratory part of a private Lab Chain or Hospital Network (pick one)?

- No
- Yes

### Results

|  | Yes | No |
| --- | --- | --- |
| Combined | 429 (47%) | 491 |
| Government hospital laboratory | 97 (24%) | 304 |
| Private hospital laboratory | 194 (66%) | 102 |
| Commercial laboratory | 138 (62%) | 85 |

## Item 04 - Patients per day

### Prompt

How many sample request forms (patients) per day is your lab processing?

{Slider from ≤ 100 to ≥ 20000}

### Results

|  | n | Max. | 3rd Qu. | Mean | SD | Median | IQR | 1st Qu. | Min. |
| --- | --- | --- | --- | --- | --- | --- | --- | --- | --- |
| Combined | 920 | 20000 | 1500 | 1670 | 3206 | 600 | 1200 | 300 | 100 |
| Government hospital laboratory | 401 | 20000 | 2000 | 2002 | 3532 | 800 | 1600 | 400 | 100 |
| Private hospital laboratory | 296 | 20000 | 1000 | 1040 | 2056 | 400 | 800 | 200 | 100 |
| Commercial laboratory | 223 | 20000 | 1650 | 1910 | 3698 | 500 | 1450 | 200 | 100 |

## Item 05 - Certification / Accreditation

### Prompt

Does your laboratory have, or plan to have any **international** accreditation (pick one for each of the following)?

- ISO 15189
- ISO 9000 Series
- ISO 14000 Series
- ISO 17025
- CAP (College of American Pathologists)
- JCIA (Joint Commission International Accreditation)

### Results

#### Combined

|  | Currently have | Plan to have in the next 12 months | Do not have |
| --- | --- | --- | --- |
| ISO 15189 | 460 (50%) | 122 | 338 |
| ISO 9000 Series | 124 (13%) | 37 | 759 |
| ISO 14000 Series | 27 (3%) | 12 | 881 |
| ISO 17025 | 33 (4%) | 9 | 878 |
| CAP | 74 (8%) | 73 | 773 |
| JCIA | 99 (11%) | 41 | 780 |

#### Government hospital laboratory

|  | Currently have | Plan to have in the next 12 months | Do not have |
| --- | --- | --- | --- |
| ISO 15189 | 159 (40%) | 61 | 181 |
| ISO 9000 Series | 56 (14%) | 21 | 324 |
| ISO 14000 Series | 15 (4%) | 5 | 381 |
| ISO 17025 | 14 (3%) | 6 | 381 |
| CAP | 29 (7%) | 26 | 346 |
| JCIA | 39 (10%) | 13 | 349 |

#### Private hospital laboratory

|  | Currently have | Plan to have in the next 12 months | Do not have |
| --- | --- | --- | --- |
| ISO 15189 | 142 (48%) | 37 | 117 |
| ISO 9000 Series | 25 (8%) | 9 | 262 |
| ISO 14000 Series | 7 (2%) | 1 | 288 |
| ISO 17025 | 6 (2%) | 0 | 290 |
| CAP | 27 (9%) | 27 | 242 |
| JCIA | 54 (18%) | 23 | 219 |

#### Commercial laboratory

|  | Currently have | Plan to have in the next 12 months | Do not have |
| --- | --- | --- | --- |
| ISO 15189 | 159 (71%) | 24 | 40 |
| ISO 9000 Series | 43 (19%) | 7 | 173 |
| ISO 14000 Series | 5 (2%) | 6 | 212 |
| ISO 17025 | 13 (6%) | 3 | 207 |
| CAP | 18 (8%) | 20 | 185 |
| JCIA | 6 (3%) | 5 | 212 |

## Item 06 - External Quality Assessment

### Prompt

Does your laboratory participate, or plan to participate in any of the following external quality control (ECQ) programs (pick one for each of the following)?

- CAP (College of American Pathologists)
- EQAS from Biorad
- RIQAS EQA from Randox
- MLE (Medical Laboratory Evaluation by American College of Physicians)
- NEQAS
- WeQAS
- ProBioQual

### Results

#### Combined

|  | Currently participate | Plan to participate in the next 12 months | Do not participate |
| --- | --- | --- | --- |
| CAP | 176 (19%) | 36 | 708 |
| EQAS | 325 (35%) | 28 | 567 |
| RIQAS | 341 (37%) | 14 | 565 |
| MLE | 16 (2%) | 6 | 898 |
| NEQAS | 110 (12%) | 7 | 803 |
| WeQAS | 16 (2%) | 3 | 901 |
| ProBioQual | 46 (5%) | 3 | 871 |

#### Government hospital laboratory

|  | Currently participate | Plan to participate in the next 12 months | Do not participate |
| --- | --- | --- | --- |
| CAP | 68 (17%) | 13 | 320 |
| EQAS | 146 (36%) | 9 | 246 |
| RIQAS | 132 (33%) | 7 | 262 |
| MLE | 4 (1%) | 1 | 396 |
| NEQAS | 66 (16%) | 4 | 331 |
| WeQAS | 13 (3%) | 1 | 387 |
| ProBioQual | 18 (4%) | 2 | 381 |

#### Private hospital laboratory

|  | Currently participate | Plan to participate in the next 12 months | Do not participate |
| --- | --- | --- | --- |
| CAP | 76 (26%) | 8 | 212 |
| EQAS | 97 (33%) | 12 | 187 |
| RIQAS | 104 (35%) | 3 | 189 |
| MLE | 8 (3%) | 1 | 287 |
| NEQAS | 27 (9%) | 1 | 268 |
| WeQAS | 3 (1%) | 1 | 292 |
| ProBioQual | 3 (1%) | 0 | 293 |

#### Commercial laboratory

|  | Currently participate | Plan to participate in the next 12 months | Do not participate |
| --- | --- | --- | --- |
| CAP | 32 (14%) | 15 | 176 |
| EQAS | 82 (37%) | 7 | 134 |
| RIQAS | 105 (47%) | 4 | 114 |
| MLE | 4 (2%) | 4 | 215 |
| NEQAS | 17 (8%) | 2 | 204 |
| WeQAS | 0 (0%) | 1 | 222 |
| ProBioQual | 25 (11%) | 1 | 197 |

## Item 07 - Practices / Tools

### Prompt

Please indicate which of the following best practices or tools are used in your laboratory (pick one for each of the following):

- LEAN Six Sigma
- Clinician satisfaction survey
- Employee satisfaction survey
- Patient satisfaction survey
- Continuous training/development program for employees
- Return on investment (e.g. TVO, TCO)
- IFCC quality indicators
- Incident reporting
- Internal audits to monitor
- lab performance
- Peer comparison/benchmarking

### Results

#### Combined

|  | Currently use | Plan to use in the next 12 months | Do not use |
| --- | --- | --- | --- |
| LEAN Six Sigma | 238 (26%) | 145 | 537 |
| Clinician satisfaction survey | 621 (68%) | 51 | 248 |
| Employee satisfaction survey | 623 (68%) | 40 | 257 |
| Patient satisfaction survey | 675 (73%) | 38 | 207 |
| Continuous training/development program for employees | 777 (84%) | 31 | 112 |
| Return on investment (e.g. TVO, TCO) | 168 (18%) | 81 | 671 |
| IFCC quality indicators | 289 (31%) | 61 | 570 |
| Incident reporting | 778 (85%) | 16 | 126 |
| Internal audits to monitor lab performance | 732 (80%) | 35 | 153 |
| Peer comparison/benchmarking | 550 (60%) | 53 | 317 |

#### Government hospital laboratory

|  | Currently use | Plan to use in the next 12 months | Do not use |
| --- | --- | --- | --- |
| LEAN Six Sigma | 103 (26%) | 63 | 235 |
| Clinician satisfaction survey | 229 (57%) | 41 | 131 |
| Employee satisfaction survey | 230 (57%) | 27 | 144 |
| Patient satisfaction survey | 235 (59%) | 29 | 137 |
| Continuous training/development program for employees | 322 (80%) | 21 | 58 |
| Return on investment (e.g. TVO, TCO) | 58 (14%) | 31 | 312 |
| IFCC quality indicators | 127 (32%) | 24 | 250 |
| Incident reporting | 337 (84%) | 9 | 55 |
| Internal audits to monitor lab performance | 307 (77%) | 15 | 79 |
| Peer comparison/benchmarking | 213 (53%) | 26 | 162 |

#### Private hospital laboratory

|  | Currently use | Plan to use in the next 12 months | Do not use |
| --- | --- | --- | --- |
| LEAN Six Sigma | 76 (26%) | 56 | 164 |
| Clinician satisfaction survey | 227 (77%) | 5 | 64 |
| Employee satisfaction survey | 241 (81%) | 7 | 48 |
| Patient satisfaction survey | 249 (84%) | 4 | 43 |
| Continuous training/development program for employees | 255 (86%) | 6 | 35 |
| Return on investment (e.g. TVO, TCO) | 54 (18%) | 25 | 217 |
| IFCC quality indicators | 82 (28%) | 21 | 193 |
| Incident reporting | 260 (88%) | 4 | 32 |
| Internal audits to monitor lab performance | 239 (81%) | 12 | 45 |
| Peer comparison/benchmarking | 185 (62%) | 14 | 97 |

#### Commercial laboratory

|  | Currently use | Plan to use in the next 12 months | Do not use |
| --- | --- | --- | --- |
| LEAN Six Sigma | 59 (26%) | 26 | 138 |
| Clinician satisfaction survey | 165 (74%) | 5 | 53 |
| Employee satisfaction survey | 152 (68%) | 6 | 65 |
| Patient satisfaction survey | 191 (86%) | 5 | 27 |
| Continuous training/development program for employees | 200 (90%) | 4 | 19 |
| Return on investment (e.g. TVO, TCO) | 56 (25%) | 25 | 142 |
| IFCC quality indicators | 80 (36%) | 16 | 127 |
| Incident reporting | 181 (81%) | 3 | 39 |
| Internal audits to monitor lab performance | 186 (83%) | 8 | 29 |
| Peer comparison/benchmarking | 152 (68%) | 13 | 58 |

## Item 08 - Effects of COVID-19

### Prompt

Did the COVID-19 pandemic changed how your laboratory operates in the following areas (pick one for each of the following)?

- Providing stronger guidance how to take samples correctly
- Controlling quality in an appropriate and visible way
- Labelling samples correctly so that the patient can be correctly identified
- Delivering results within a clinically meaningful timeframe
- Making results visible to all those who need to see them
- Providing advice and support on interpretations and appropriate responses to results
- Ability of computer system and software to exchange and to make use of information across locations

### Results

#### Combined

|  | Changes established | Plan to make changes in the next 12 months | No changes |
| --- | --- | --- | --- |
| Providing stronger guidance how to take samples correctly | 574 (62%) | 6 | 340 |
| Controlling quality in an appropriate and visible way | 442 (48%) | 9 | 469 |
| Labelling samples correctly so that the patient can be correctly identified | 428 (47%) | 4 | 488 |
| Delivering results within a clinically meaningful timeframe | 476 (52%) | 3 | 441 |
| Making results visible to all those who need to see them | 433 (47%) | 17 | 470 |
| Providing advice and support on interpretations and appropriate responses to results | 448 (49%) | 11 | 461 |
| Ability of computer system and software to exchange and to make use of information across locations | 408 (44%) | 40 | 472 |

#### Government hospital laboratory

|  | Changes established | Plan to make changes in the next 12 months | No changes |
| --- | --- | --- | --- |
| Providing stronger guidance how to take samples correctly | 236 (59%) | 4 | 161 |
| Controlling quality in an appropriate and visible way | 175 (44%) | 5 | 221 |
| Labelling samples correctly so that the patient can be correctly identified | 174 (43%) | 1 | 226 |
| Delivering results within a clinically meaningful timeframe | 196 (49%) | 2 | 203 |
| Making results visible to all those who need to see them | 177 (44%) | 8 | 216 |
| Providing advice and support on interpretations and appropriate responses to results | 180 (45%) | 5 | 216 |
| Ability of computer system and software to exchange and to make use of information across locations | 166 (41%) | 17 | 218 |

#### Private hospital laboratory

|  | Changes established | Plan to make changes in the next 12 months | No changes |
| --- | --- | --- | --- |
| Providing stronger guidance how to take samples correctly | 218 (74%) | 2 | 76 |
| Controlling quality in an appropriate and visible way | 168 (57%) | 3 | 125 |
| Labelling samples correctly so that the patient can be correctly identified | 171 (58%) | 0 | 125 |
| Delivering results within a clinically meaningful timeframe | 180 (61%) | 0 | 116 |
| Making results visible to all those who need to see them | 154 (52%) | 5 | 137 |
| Providing advice and support on interpretations and appropriate responses to results | 163 (55%) | 5 | 128 |
| Ability of computer system and software to exchange and to make use of information across locations | 150 (51%) | 15 | 131 |

#### Commercial laboratory

|  | Changes established | Plan to make changes in the next 12 months | No changes |
| --- | --- | --- | --- |
| Providing stronger guidance how to take samples correctly | 120 (54%) | 0 | 103 |
| Controlling quality in an appropriate and visible way | 99 (44%) | 1 | 123 |
| Labelling samples correctly so that the patient can be correctly identified | 83 (37%) | 3 | 137 |
| Delivering results within a clinically meaningful timeframe | 100 (45%) | 1 | 122 |
| Making results visible to all those who need to see them | 102 (46%) | 4 | 117 |
| Providing advice and support on interpretations and appropriate responses to results | 105 (47%) | 1 | 117 |
| Ability of computer system and software to exchange and to make use of information across locations | 92 (41%) | 8 | 123 |

## Item 09 - Dedicated laboratories

### Prompt

In addition to the core laboratory, do you have any other dedicated laboratories in your institution/organization (pick one or more of the following)?

- None
- Emergency/STAT lab
- Serology lab
- Coagulation lab
- Urinanalysis lab
- Molecular lab
- HPLC lab
- Microbiology lab
- Histopathology lab

### Results

#### Combined

|  | Yes | No |
| --- | --- | --- |
| Emergency/STAT lab | 229 (25%) | 691 |
| Serology lab | 370 (40%) | 550 |
| Coagulation lab | 371 (40%) | 549 |
| Urinanalysis lab | 387 (42%) | 533 |
| Molecular lab | 429 (47%) | 491 |
| HPLC lab | 153 (17%) | 767 |
| Microbiology lab | 498 (54%) | 422 |
| Histopathology lab | 349 (38%) | 571 |
| None | 262 (28%) | 658 |

#### Government hospital laboratory

|  | Yes | No |
| --- | --- | --- |
| Emergency/STAT lab | 126 (31%) | 275 |
| Serology lab | 182 (45%) | 219 |
| Coagulation lab | 188 (47%) | 213 |
| Urinanalysis lab | 182 (45%) | 219 |
| Molecular lab | 190 (47%) | 211 |
| HPLC lab | 82 (20%) | 319 |
| Microbiology lab | 245 (61%) | 156 |
| Histopathology lab | 183 (46%) | 218 |
| None | 89 (22%) | 312 |

#### Private hospital laboratory

|  | Yes | No |
| --- | --- | --- |
| Emergency/STAT lab | 54 (18%) | 242 |
| Serology lab | 96 (32%) | 200 |
| Coagulation lab | 94 (32%) | 202 |
| Urinanalysis lab | 109 (37%) | 187 |
| Molecular lab | 137 (46%) | 159 |
| HPLC lab | 24 (8%) | 272 |
| Microbiology lab | 148 (50%) | 148 |
| Histopathology lab | 100 (34%) | 196 |
| None | 102 (34%) | 194 |

#### Commercial laboratory

|  | Yes | No |
| --- | --- | --- |
| Emergency/STAT lab | 49 (22%) | 174 |
| Serology lab | 92 (41%) | 131 |
| Coagulation lab | 89 (40%) | 134 |
| Urinanalysis lab | 96 (43%) | 127 |
| Molecular lab | 102 (46%) | 121 |
| HPLC lab | 47 (21%) | 176 |
| Microbiology lab | 105 (47%) | 118 |
| Histopathology lab | 66 (30%) | 157 |
| None | 71 (32%) | 152 |

## Item 10 - Pre-laboratory indicators

### Prompt

What pre lab indicators do you monitor (pick one for each of the following)?

- Transport duration
- Transport temperature
- Rough handling during transport
- Missing tubes
- Labelling problems
- Use of inappropriate sample
- containers
- Sample volume
- Sample quality (Haemolytic/Icteric/Lipaemic)
- Sample quality vs time from collection
- Average of normal potassium vs time from collection
- Impact of light on sample quality

### Results

#### Combined

|  | Currently monitor | Plan to monitor in the next 12 months | Do not monitor |
| --- | --- | --- | --- |
| Transport duration | 631 (69%) | 60 | 229 |
| Transport temperature | 615 (67%) | 62 | 243 |
| Rough handling during transport | 448 (49%) | 54 | 418 |
| Missing tubes | 768 (83%) | 30 | 122 |
| Labelling problems | 809 (88%) | 18 | 93 |
| Use of inappropriate sample containers | 802 (87%) | 11 | 107 |
| Sample volume | 835 (91%) | 16 | 69 |
| Sample quality (Haemolytic/Icteric/Lipaemic) | 860 (93%) | 14 | 46 |
| Sample quality vs time from collection | 632 (69%) | 37 | 251 |
| Average of normal potassium vs time from collection | 338 (37%) | 53 | 529 |
| Impact of light on sample quality | 381 (41%) | 43 | 496 |

#### Government hospital laboratory

|  | Currently monitor | Plan to monitor in the next 12 months | Do not monitor |
| --- | --- | --- | --- |
| Transport duration | 228 (57%) | 35 | 138 |
| Transport temperature | 212 (53%) | 42 | 147 |
| Rough handling during transport | 156 (39%) | 31 | 214 |
| Missing tubes | 317 (79%) | 19 | 65 |
| Labelling problems | 336 (84%) | 15 | 50 |
| Use of inappropriate sample containers | 339 (85%) | 7 | 55 |
| Sample volume | 357 (89%) | 9 | 35 |
| Sample quality (Haemolytic/Icteric/Lipaemic) | 373 (93%) | 7 | 21 |
| Sample quality vs time from collection | 255 (64%) | 24 | 122 |
| Average of normal potassium vs time from collection | 128 (32%) | 27 | 246 |
| Impact of light on sample quality | 141 (35%) | 22 | 238 |

#### Private hospital laboratory

|  | Currently monitor | Plan to monitor in the next 12 months | Do not monitor |
| --- | --- | --- | --- |
| Transport duration | 212 (72%) | 19 | 65 |
| Transport temperature | 215 (73%) | 15 | 66 |
| Rough handling during transport | 169 (57%) | 13 | 114 |
| Missing tubes | 243 (82%) | 10 | 43 |
| Labelling problems | 264 (89%) | 3 | 29 |
| Use of inappropriate sample containers | 261 (88%) | 4 | 31 |
| Sample volume | 270 (91%) | 5 | 21 |
| Sample quality (Haemolytic/Icteric/Lipaemic) | 275 (93%) | 6 | 15 |
| Sample quality vs time from collection | 200 (68%) | 6 | 90 |
| Average of normal potassium vs time from collection | 96 (32%) | 14 | 186 |
| Impact of light on sample quality | 121 (41%) | 10 | 165 |

#### Commercial laboratory

|  | Currently monitor | Plan to monitor in the next 12 months | Do not monitor |
| --- | --- | --- | --- |
| Transport duration | 191 (86%) | 6 | 26 |
| Transport temperature | 188 (84%) | 5 | 30 |
| Rough handling during transport | 123 (55%) | 10 | 90 |
| Missing tubes | 208 (93%) | 1 | 14 |
| Labelling problems | 209 (94%) | 0 | 14 |
| Use of inappropriate sample containers | 202 (91%) | 0 | 21 |
| Sample volume | 208 (93%) | 2 | 13 |
| Sample quality (Haemolytic/Icteric/Lipaemic) | 212 (95%) | 1 | 10 |
| Sample quality vs time from collection | 177 (79%) | 7 | 39 |
| Average of normal potassium vs time from collection | 114 (51%) | 12 | 97 |
| Impact of light on sample quality | 119 (53%) | 11 | 93 |

# Items 11-20

## Item 11 - Key Performance Indicators

### Prompt

Does your laboratory measure defined Key Performance Indicators (KPIs) (pick one for each of the following)?

- Turn around time (TAT)
- Employee productivity (e.g. Samples per FTE)
- Work space utilization (e.g. Tests per square meter)
- Total consumable waste
- Expired reagent stock
- Instrument noise levels
- Systems Uptime/Downtime
- Time spent on technical and medical validation of results
- Rerun Rates
- Specimen acceptance/rejection rate

### Results

#### Combined

|  | Currently measure | Plan to measure in the next 12 months | Do not measure |
| --- | --- | --- | --- |
| Turn around time (TAT) | 820 (89%) | 27 | 73 |
| Employee productivity (e.g. Samples per FTE) | 346 (38%) | 95 | 479 |
| Work space utilization (e.g. Tests per square meter) | 188 (20%) | 97 | 635 |
| Total consumable waste | 411 (45%) | 74 | 435 |
| Expired reagent stock | 686 (75%) | 49 | 185 |
| Instrument noise levels | 269 (29%) | 76 | 575 |
| Systems Uptime/Downtime | 486 (53%) | 64 | 370 |
| Time spent on technical and medical validation of results | 382 (42%) | 80 | 458 |
| Rerun Rates | 481 (52%) | 84 | 355 |
| Specimen acceptance/rejection rate | 606 (66%) | 68 | 246 |

#### Government hospital laboratory

|  | Currently measure | Plan to measure in the next 12 months | Do not measure |
| --- | --- | --- | --- |
| Turn around time (TAT) | 346 (86%) | 14 | 41 |
| Employee productivity (e.g. Samples per FTE) | 128 (32%) | 38 | 235 |
| Work space utilization (e.g. Tests per square meter) | 65 (16%) | 38 | 298 |
| Total consumable waste | 132 (33%) | 34 | 235 |
| Expired reagent stock | 267 (67%) | 30 | 104 |
| Instrument noise levels | 125 (31%) | 31 | 245 |
| Systems Uptime/Downtime | 194 (48%) | 36 | 171 |
| Time spent on technical and medical validation of results | 151 (38%) | 36 | 214 |
| Rerun Rates | 179 (45%) | 44 | 178 |
| Specimen acceptance/rejection rate | 241 (60%) | 29 | 131 |

#### Private hospital laboratory

|  | Currently measure | Plan to measure in the next 12 months | Do not measure |
| --- | --- | --- | --- |
| Turn around time (TAT) | 279 (94%) | 5 | 12 |
| Employee productivity (e.g. Samples per FTE) | 115 (39%) | 34 | 147 |
| Work space utilization (e.g. Tests per square meter) | 59 (20%) | 34 | 203 |
| Total consumable waste | 144 (49%) | 23 | 129 |
| Expired reagent stock | 228 (77%) | 12 | 56 |
| Instrument noise levels | 73 (25%) | 21 | 202 |
| Systems Uptime/Downtime | 147 (50%) | 16 | 133 |
| Time spent on technical and medical validation of results | 113 (38%) | 31 | 152 |
| Rerun Rates | 171 (58%) | 22 | 103 |
| Specimen acceptance/rejection rate | 199 (67%) | 29 | 68 |

#### Commercial laboratory

|  | Currently measure | Plan to measure in the next 12 months | Do not measure |
| --- | --- | --- | --- |
| Turn around time (TAT) | 195 (87%) | 8 | 20 |
| Employee productivity (e.g. Samples per FTE) | 103 (46%) | 23 | 97 |
| Work space utilization (e.g. Tests per square meter) | 64 (29%) | 25 | 134 |
| Total consumable waste | 135 (61%) | 17 | 71 |
| Expired reagent stock | 191 (86%) | 7 | 25 |
| Instrument noise levels | 71 (32%) | 24 | 128 |
| Systems Uptime/Downtime | 145 (65%) | 12 | 66 |
| Time spent on technical and medical validation of results | 118 (53%) | 13 | 92 |
| Rerun Rates | 131 (59%) | 18 | 74 |
| Specimen acceptance/rejection rate | 166 (74%) | 10 | 47 |

## Item 12 - Turn-around time (TAT)

### Prompt

Which of the following Turn- Around-Times (TAT) displayed below does your laboratory monitor (pick one for each of the following)?

- Clinician Expectation Time (from order generation to access of the results by the clinician/physician)
- Sample to Result TAT (from sampling to result release)
- Lab TAT (from sample reception to result release)
- Pre-Lab TAT (from sampling to sample reception in the lab)
- Pre-analytical TAT (from sample reception to start of the analysis)
- Analytical TAT (from start of the analysis to result completion)
- Post-Analytic TAT (from result completion to result release)

### Results

#### Combined

|  | Currently monitor | Plan to monitor in the next 12 months | Do not monitor |
| --- | --- | --- | --- |
| Clinician Expectation Time (from order generation to access of the results by the clinician/physician) | 365 (40%) | 59 | 496 |
| Sample to Result TAT (from sampling to result release) | 553 (60%) | 54 | 313 |
| Lab TAT (from sample reception to result release) | 803 (87%) | 21 | 96 |
| Pre-Lab TAT (from sampling to sample reception in the lab) | 398 (43%) | 93 | 429 |
| Pre-analytical TAT (from sample reception to start of the analysis) | 470 (51%) | 71 | 379 |
| Analytical TAT (from start of the analysis to result completion) | 563 (61%) | 43 | 314 |
| Post-Analytic TAT (from result completion to result release) | 470 (51%) | 61 | 389 |

#### Government hospital laboratory

|  | Currently monitor | Plan to monitor in the next 12 months | Do not monitor |
| --- | --- | --- | --- |
| Clinician Expectation Time (from order generation to access of the results by the clinician/physician) | 145 (36%) | 25 | 231 |
| Sample to Result TAT (from sampling to result release) | 231 (58%) | 21 | 149 |
| Lab TAT (from sample reception to result release) | 345 (86%) | 8 | 48 |
| Pre-Lab TAT (from sampling to sample reception in the lab) | 163 (41%) | 45 | 193 |
| Pre-analytical TAT (from sample reception to start of the analysis) | 191 (48%) | 32 | 178 |
| Analytical TAT (from start of the analysis to result completion) | 225 (56%) | 20 | 156 |
| Post-Analytic TAT (from result completion to result release) | 195 (49%) | 22 | 184 |

#### Private hospital laboratory

|  | Currently monitor | Plan to monitor in the next 12 months | Do not monitor |
| --- | --- | --- | --- |
| Clinician Expectation Time (from order generation to access of the results by the clinician/physician) | 102 (34%) | 23 | 171 |
| Sample to Result TAT (from sampling to result release) | 158 (53%) | 18 | 120 |
| Lab TAT (from sample reception to result release) | 260 (88%) | 7 | 29 |
| Pre-Lab TAT (from sampling to sample reception in the lab) | 92 (31%) | 29 | 175 |
| Pre-analytical TAT (from sample reception to start of the analysis) | 141 (48%) | 22 | 133 |
| Analytical TAT (from start of the analysis to result completion) | 174 (59%) | 13 | 109 |
| Post-Analytic TAT (from result completion to result release) | 140 (47%) | 23 | 133 |

#### Commercial laboratory

|  | Currently monitor | Plan to monitor in the next 12 months | Do not monitor |
| --- | --- | --- | --- |
| Clinician Expectation Time (from order generation to access of the results by the clinician/physician) | 118 (53%) | 11 | 94 |
| Sample to Result TAT (from sampling to result release) | 164 (74%) | 15 | 44 |
| Lab TAT (from sample reception to result release) | 198 (89%) | 6 | 19 |
| Pre-Lab TAT (from sampling to sample reception in the lab) | 143 (64%) | 19 | 61 |
| Pre-analytical TAT (from sample reception to start of the analysis) | 138 (62%) | 17 | 68 |
| Analytical TAT (from start of the analysis to result completion) | 164 (74%) | 10 | 49 |
| Post-Analytic TAT (from result completion to result release) | 135 (61%) | 16 | 72 |

## Item 13 - Target laboratory TAT

### Prompt

What is the target **Lab TAT** for ROUTINE and URGENT samples in the following laboratory sections (in hours)?

- Clinical Chemistry (i.e. Biochemistry, Turbidimetric Assays)
- Immunoassays incl. serology (i.e. Hormones-, Tumor-, Cardiac- Markers, SARS-CoV-2)
- Haematology
- Coagulation
- Urinalysis
- Routine Molecular Diagnostics (i.e. HPV, HIV, HBV, CT/NG, SARS-CoV-2)

### Results

#### Combined

##### Routine

| Specialty | n | Max. | 3rd Qu. | Mean | SD | Median | IQR | 1st Qu. | Min. |
| --- | --- | --- | --- | --- | --- | --- | --- | --- | --- |
| Clinical chemistry | 878 | 168 | 8 | 9.1 | 17.6 | 3 | 6 | 2 | 0.5 |
| Immunoassays incl. serology | 837 | 168 | 8 | 12.7 | 23.4 | 4 | 6 | 2 | 0.5 |
| Haematology | 832 | 168 | 6 | 7.5 | 14.2 | 2 | 5 | 1 | 0.5 |
| Coagulation | 770 | 168 | 6 | 7.5 | 14.5 | 2 | 5 | 1 | 0.5 |
| Urinalysis | 785 | 168 | 8 | 8.0 | 14.9 | 3 | 7 | 1 | 0.5 |
| Routine molecular diagnostics | 499 | 168 | 24 | 24.0 | 32.8 | 12 | 20 | 4 | 0.5 |

##### Urgent

| Specialty | n | Max. | 3rd Qu. | Mean | SD | Median | IQR | 1st Qu. | Min. |
| --- | --- | --- | --- | --- | --- | --- | --- | --- | --- |
| Clinical chemistry | 879 | 120 | 1 | 3.0 | 9.6 | 1 | 0.0 | 1.0 | 0.5 |
| Immunoassays incl. serology | 792 | 120 | 2 | 4.1 | 11.6 | 1 | 1.0 | 1.0 | 0.5 |
| Haematology | 836 | 120 | 1 | 2.6 | 8.7 | 1 | 0.5 | 0.5 | 0.5 |
| Coagulation | 779 | 120 | 1 | 3.0 | 9.5 | 1 | 0.5 | 0.5 | 0.5 |
| Urinalysis | 753 | 75 | 1 | 3.0 | 8.4 | 1 | 0.5 | 0.5 | 0.5 |
| Routine molecular diagnostics | 425 | 168 | 12 | 11.4 | 22.5 | 4 | 10.0 | 2.0 | 0.5 |

#### Government hospital laboratory

##### Routine

| Specialty | n | Max. | 3rd Qu. | Mean | SD | Median | IQR | 1st Qu. | Min. |
| --- | --- | --- | --- | --- | --- | --- | --- | --- | --- |
| Clinical chemistry | 380 | 168 | 8 | 10.0 | 20.7 | 3 | 6 | 2 | 0.5 |
| Immunoassays incl. serology | 362 | 168 | 12 | 15.5 | 29.4 | 4 | 10 | 2 | 0.5 |
| Haematology | 339 | 168 | 6 | 8.1 | 17.8 | 2 | 5 | 1 | 0.5 |
| Coagulation | 314 | 168 | 6 | 8.6 | 18.6 | 2 | 5 | 1 | 0.5 |
| Urinalysis | 330 | 168 | 8 | 9.5 | 18.9 | 3 | 7 | 1 | 0.5 |
| Routine molecular diagnostics | 203 | 168 | 24 | 26.8 | 39.5 | 12 | 20 | 4 | 0.5 |

##### Urgent

| Specialty | n | Max. | 3rd Qu. | Mean | SD | Median | IQR | 1st Qu. | Min. |
| --- | --- | --- | --- | --- | --- | --- | --- | --- | --- |
| Clinical chemistry | 385 | 120 | 1 | 3.8 | 12.3 | 1.0 | 0.5 | 0.5 | 0.5 |
| Immunoassays incl. serology | 356 | 120 | 2 | 4.7 | 13.5 | 1.0 | 1.0 | 1.0 | 0.5 |
| Haematology | 347 | 120 | 1 | 3.2 | 11.2 | 1.0 | 0.5 | 0.5 | 0.5 |
| Coagulation | 319 | 120 | 1 | 4.0 | 12.7 | 1.0 | 0.5 | 0.5 | 0.5 |
| Urinalysis | 313 | 75 | 1 | 3.4 | 9.9 | 1.0 | 0.5 | 0.5 | 0.5 |
| Routine molecular diagnostics | 180 | 168 | 8 | 11.3 | 23.2 | 2.5 | 7.0 | 1.0 | 0.5 |

#### Private hospital laboratory

##### Routine

| Specialty | n | Max. | 3rd Qu. | Mean | SD | Median | IQR | 1st Qu. | Min. |
| --- | --- | --- | --- | --- | --- | --- | --- | --- | --- |
| Clinical chemistry | 289 | 168 | 4 | 7.3 | 17.2 | 2 | 2 | 2 | 0.5 |
| Immunoassays incl. serology | 275 | 168 | 6 | 10.0 | 19.2 | 3 | 4 | 2 | 0.5 |
| Haematology | 286 | 120 | 4 | 5.7 | 11.8 | 2 | 3 | 1 | 0.5 |
| Coagulation | 271 | 120 | 4 | 5.7 | 11.8 | 2 | 3 | 1 | 0.5 |
| Urinalysis | 273 | 100 | 4 | 6.3 | 11.9 | 2 | 3 | 1 | 0.5 |
| Routine molecular diagnostics | 170 | 168 | 24 | 23.8 | 32.1 | 18 | 21 | 3 | 0.5 |

##### Urgent

| Specialty | n | Max. | 3rd Qu. | Mean | SD | Median | IQR | 1st Qu. | Min. |
| --- | --- | --- | --- | --- | --- | --- | --- | --- | --- |
| Clinical chemistry | 287 | 60 | 1 | 2.8 | 8.6 | 1 | 0.5 | 0.5 | 0.5 |
| Immunoassays incl. serology | 240 | 90 | 2 | 4.4 | 12.7 | 1 | 1.0 | 1.0 | 0.5 |
| Haematology | 280 | 83 | 1 | 2.6 | 8.2 | 1 | 0.5 | 0.5 | 0.5 |
| Coagulation | 266 | 60 | 1 | 2.8 | 8.1 | 1 | 0.5 | 0.5 | 0.5 |
| Urinalysis | 265 | 60 | 1 | 3.0 | 8.8 | 1 | 0.5 | 0.5 | 0.5 |
| Routine molecular diagnostics | 130 | 168 | 12 | 13.5 | 27.4 | 3 | 10.0 | 2.0 | 0.5 |

#### Commercial laboratory

##### Routine

| Specialty | n | Max. | 3rd Qu. | Mean | SD | Median | IQR | 1st Qu. | Min. |
| --- | --- | --- | --- | --- | --- | --- | --- | --- | --- |
| Clinical chemistry | 209 | 72 | 24.0 | 10.1 | 10.4 | 5 | 21.0 | 3.0 | 0.5 |
| Immunoassays incl. serology | 200 | 120 | 24.0 | 11.4 | 14.4 | 6 | 21.0 | 3.0 | 1.0 |
| Haematology | 207 | 72 | 12.0 | 9.2 | 9.8 | 5 | 10.0 | 2.0 | 0.5 |
| Coagulation | 185 | 24 | 12.0 | 8.3 | 8.7 | 4 | 10.0 | 2.0 | 0.5 |
| Urinalysis | 182 | 72 | 8.8 | 7.9 | 9.5 | 4 | 6.8 | 2.0 | 0.5 |
| Routine molecular diagnostics | 126 | 120 | 24.0 | 19.8 | 18.7 | 12 | 17.8 | 6.2 | 0.5 |

##### Urgent

| Specialty | n | Max. | 3rd Qu. | Mean | SD | Median | IQR | 1st Qu. | Min. |
| --- | --- | --- | --- | --- | --- | --- | --- | --- | --- |
| Clinical chemistry | 207 | 24 | 2 | 1.8 | 2.4 | 1 | 1 | 1 | 0.5 |
| Immunoassays incl. serology | 196 | 24 | 2 | 2.5 | 3.7 | 2 | 1 | 1 | 0.5 |
| Haematology | 209 | 24 | 2 | 1.7 | 2.4 | 1 | 1 | 1 | 0.5 |
| Coagulation | 194 | 24 | 2 | 1.7 | 1.9 | 1 | 1 | 1 | 0.5 |
| Urinalysis | 175 | 24 | 2 | 2.1 | 3.6 | 1 | 1 | 1 | 0.5 |
| Routine molecular diagnostics | 115 | 96 | 12 | 9.1 | 12.9 | 5 | 10 | 2 | 0.5 |

## Item 14 - Tools to monitor TAT

### Prompt

Which tools do you use to monitor your TAT (pick one)?

### Results

|  | Comprehensive/Dedicated | Spreadsheet | None |
| --- | --- | --- | --- |
| Combined | 367 | 432 | 121 |
| Government hospital laboratory | 144 | 196 | 61 |
| Private hospital laboratory | 139 | 136 | 21 |
| Commercial laboratory | 84 | 100 | 39 |

## Item 15 - Electronic ordering

### Prompt

What percentage of requests are ordered electronically and **don’t** require a paper order form that needs to be registered manually into your LIS?

{Slider from 0% to 100%}

### Results

|  | n | Max. | 3rd Qu. | Mean | SD | Median | IQR | 1st Qu. | Min. |
| --- | --- | --- | --- | --- | --- | --- | --- | --- | --- |
| Combined | 920 | 100 | 100 | 57 | 41 | 70 | 90 | 10 | 0 |
| Government hospital laboratory | 401 | 100 | 100 | 65 | 40 | 90 | 70 | 30 | 0 |
| Private hospital laboratory | 296 | 100 | 100 | 61 | 39 | 70 | 80 | 20 | 0 |
| Commercial laboratory | 223 | 100 | 80 | 37 | 41 | 10 | 80 | 0 | 0 |

## Item 16 - Verification / Validation

### Prompt

What is the target Lab TAT for ROUTINE and URGENT samples in the following laboratory sections (in hours)?

- Clinical Chemistry (i.e. Biochemistry, Turbidimetric Assays)
- Immunoassays incl. serology (i.e. Hormones-, Tumor-, Cardiac- Markers, SARS-CoV-2)
- Haematology
- Coagulation
- Urinalysis
- Routine Molecular Diagnostics (i.e. HPV, HIV, HBV, CT/NG, SARS-CoV-2)

### Results

#### Combined

##### Technical auto-verification rate in %

| Specialty | n | Max. | 3rd Qu. | Mean | SD | Median | IQR | 1st Qu. | Min. |
| --- | --- | --- | --- | --- | --- | --- | --- | --- | --- |
| Clinical chemistry | 908 | 100 | 70 | 29.8 | 38.9 | 0 | 70 | 0 | 0 |
| Immunoassays incl. serology | 886 | 100 | 60 | 27.0 | 37.8 | 0 | 60 | 0 | 0 |
| Haematology | 870 | 100 | 70 | 27.8 | 38.6 | 0 | 70 | 0 | 0 |
| Coagulation | 820 | 100 | 50 | 23.5 | 37.4 | 0 | 50 | 0 | 0 |
| Urinalysis | 826 | 100 | 50 | 22.8 | 37.0 | 0 | 50 | 0 | 0 |
| Routine molecular diagnostics | 592 | 100 | 0 | 16.5 | 32.8 | 0 | 0 | 0 | 0 |

##### Clinical auto-validation rate in %

| Specialty | n | Max. | 3rd Qu. | Mean | SD | Median | IQR | 1st Qu. | Min. |
| --- | --- | --- | --- | --- | --- | --- | --- | --- | --- |
| Clinical chemistry | 907 | 100 | 0 | 11.0 | 26.2 | 0 | 0 | 0 | 0 |
| Immunoassays incl. serology | 882 | 100 | 0 | 10.4 | 25.5 | 0 | 0 | 0 | 0 |
| Haematology | 867 | 100 | 0 | 10.4 | 25.4 | 0 | 0 | 0 | 0 |
| Coagulation | 818 | 100 | 0 | 9.7 | 25.2 | 0 | 0 | 0 | 0 |
| Urinalysis | 824 | 100 | 0 | 10.1 | 25.8 | 0 | 0 | 0 | 0 |
| Routine molecular diagnostics | 591 | 100 | 0 | 8.1 | 23.6 | 0 | 0 | 0 | 0 |

#### Government hospital laboratory

##### Technical auto-verification rate in %

| Specialty | n | Max. | 3rd Qu. | Mean | SD | Median | IQR | 1st Qu. | Min. |
| --- | --- | --- | --- | --- | --- | --- | --- | --- | --- |
| Clinical chemistry | 391 | 100 | 75 | 32.3 | 39.9 | 0 | 75 | 0 | 0 |
| Immunoassays incl. serology | 381 | 100 | 70 | 29.8 | 39.2 | 0 | 70 | 0 | 0 |
| Haematology | 359 | 100 | 70 | 30.0 | 39.1 | 0 | 70 | 0 | 0 |
| Coagulation | 337 | 100 | 70 | 27.4 | 38.7 | 0 | 70 | 0 | 0 |
| Urinalysis | 352 | 100 | 60 | 26.3 | 38.5 | 0 | 60 | 0 | 0 |
| Routine molecular diagnostics | 246 | 100 | 0 | 16.6 | 32.5 | 0 | 0 | 0 | 0 |

##### Clinical auto-validation rate in %

| Specialty | n | Max. | 3rd Qu. | Mean | SD | Median | IQR | 1st Qu. | Min. |
| --- | --- | --- | --- | --- | --- | --- | --- | --- | --- |
| Clinical chemistry | 390 | 100 | 0 | 12.2 | 27.2 | 0 | 0 | 0 | 0 |
| Immunoassays incl. serology | 378 | 100 | 0 | 11.6 | 26.4 | 0 | 0 | 0 | 0 |
| Haematology | 357 | 100 | 0 | 12.1 | 26.3 | 0 | 0 | 0 | 0 |
| Coagulation | 336 | 100 | 0 | 11.6 | 26.6 | 0 | 0 | 0 | 0 |
| Urinalysis | 351 | 100 | 0 | 12.0 | 27.0 | 0 | 0 | 0 | 0 |
| Routine molecular diagnostics | 245 | 100 | 0 | 9.3 | 24.8 | 0 | 0 | 0 | 0 |

#### Private hospital laboratory

##### Technical auto-verification rate in %

| Specialty | n | Max. | 3rd Qu. | Mean | SD | Median | IQR | 1st Qu. | Min. |
| --- | --- | --- | --- | --- | --- | --- | --- | --- | --- |
| Clinical chemistry | 295 | 100 | 70.0 | 28.3 | 37.7 | 0 | 70.0 | 0 | 0 |
| Immunoassays incl. serology | 289 | 100 | 55.0 | 24.9 | 36.1 | 0 | 55.0 | 0 | 0 |
| Haematology | 291 | 100 | 67.5 | 26.5 | 37.8 | 0 | 67.5 | 0 | 0 |
| Coagulation | 283 | 100 | 10.0 | 18.3 | 33.8 | 0 | 10.0 | 0 | 0 |
| Urinalysis | 285 | 100 | 10.0 | 18.6 | 34.2 | 0 | 10.0 | 0 | 0 |
| Routine molecular diagnostics | 201 | 100 | 0.0 | 14.6 | 31.0 | 0 | 0.0 | 0 | 0 |

##### Clinical auto-validation rate in %

| Specialty | n | Max. | 3rd Qu. | Mean | SD | Median | IQR | 1st Qu. | Min. |
| --- | --- | --- | --- | --- | --- | --- | --- | --- | --- |
| Clinical chemistry | 295 | 100 | 0 | 6.5 | 21.3 | 0 | 0 | 0 | 0 |
| Immunoassays incl. serology | 288 | 100 | 0 | 6.1 | 20.2 | 0 | 0 | 0 | 0 |
| Haematology | 290 | 100 | 0 | 6.2 | 20.9 | 0 | 0 | 0 | 0 |
| Coagulation | 282 | 100 | 0 | 5.7 | 20.5 | 0 | 0 | 0 | 0 |
| Urinalysis | 284 | 100 | 0 | 5.7 | 20.4 | 0 | 0 | 0 | 0 |
| Routine molecular diagnostics | 201 | 100 | 0 | 4.3 | 17.3 | 0 | 0 | 0 | 0 |

#### Commercial laboratory

##### Technical auto-verification rate in %

| Specialty | n | Max. | 3rd Qu. | Mean | SD | Median | IQR | 1st Qu. | Min. |
| --- | --- | --- | --- | --- | --- | --- | --- | --- | --- |
| Clinical chemistry | 222 | 100 | 60.0 | 27.4 | 38.5 | 0 | 60.0 | 0 | 0 |
| Immunoassays incl. serology | 216 | 100 | 50.0 | 24.7 | 37.5 | 0 | 50.0 | 0 | 0 |
| Haematology | 220 | 100 | 52.5 | 25.8 | 38.6 | 0 | 52.5 | 0 | 0 |
| Coagulation | 200 | 100 | 50.0 | 24.4 | 39.0 | 0 | 50.0 | 0 | 0 |
| Urinalysis | 189 | 100 | 50.0 | 22.7 | 37.8 | 0 | 50.0 | 0 | 0 |
| Routine molecular diagnostics | 145 | 100 | 10.0 | 19.2 | 35.6 | 0 | 10.0 | 0 | 0 |

##### Clinical auto-validation rate in %

| Specialty | n | Max. | 3rd Qu. | Mean | SD | Median | IQR | 1st Qu. | Min. |
| --- | --- | --- | --- | --- | --- | --- | --- | --- | --- |
| Clinical chemistry | 222 | 100 | 10.0 | 14.8 | 29.5 | 0 | 10.0 | 0 | 0 |
| Immunoassays incl. serology | 216 | 100 | 6.2 | 14.1 | 29.2 | 0 | 6.2 | 0 | 0 |
| Haematology | 220 | 100 | 0.0 | 13.1 | 28.4 | 0 | 0.0 | 0 | 0 |
| Coagulation | 200 | 100 | 0.0 | 12.3 | 28.2 | 0 | 0.0 | 0 | 0 |
| Urinalysis | 189 | 100 | 0.0 | 13.2 | 29.6 | 0 | 0.0 | 0 | 0 |
| Routine molecular diagnostics | 145 | 100 | 0.0 | 11.3 | 28.1 | 0 | 0.0 | 0 | 0 |

## Item 17 - Basic IT functionalities

### Prompt

Which of the following basic functionalities do you currently use in your IT solutions? (pick one for each of the following)?

- Tracking costs per tests
- Color-coded or other types of prioritization to report results
- Age/gender related rules
- Westgard rules
- Generation of statistics reports
- Delta Checks
- Serum Index
- Checks
- QC Monitoring
- Turnaround time monitoring
- Sample archiving

### Results

#### Combined

|  | Currently use | Plan to use in the next 12 months | Do not use |
| --- | --- | --- | --- |
| Tracking costs per tests | 324 (35%) | 68 | 528 |
| Color-coded or other types of prioritization to report results | 600 (65%) | 41 | 279 |
| Age/gender related rules | 740 (80%) | 24 | 156 |
| Westgard rules | 657 (71%) | 54 | 209 |
| Generation of statistics reports | 707 (77%) | 50 | 163 |
| Delta Checks | 524 (57%) | 84 | 312 |
| Serum Index Checks | 528 (57%) | 78 | 314 |
| QC Monitoring | 724 (79%) | 61 | 135 |
| Turnaround time monitoring | 733 (80%) | 47 | 140 |
| Sample archiving | 589 (64%) | 55 | 276 |

#### Government hospital laboratory

|  | Currently use | Plan to use in the next 12 months | Do not use |
| --- | --- | --- | --- |
| Tracking costs per tests | 125 (31%) | 27 | 249 |
| Color-coded or other types of prioritization to report results | 248 (62%) | 21 | 132 |
| Age/gender related rules | 315 (79%) | 8 | 78 |
| Westgard rules | 285 (71%) | 21 | 95 |
| Generation of statistics reports | 308 (77%) | 21 | 72 |
| Delta Checks | 220 (55%) | 45 | 136 |
| Serum Index Checks | 252 (63%) | 32 | 117 |
| QC Monitoring | 316 (79%) | 27 | 58 |
| Turnaround time monitoring | 316 (79%) | 21 | 64 |
| Sample archiving | 259 (65%) | 25 | 117 |

#### Private hospital laboratory

|  | Currently use | Plan to use in the next 12 months | Do not use |
| --- | --- | --- | --- |
| Tracking costs per tests | 93 (31%) | 22 | 181 |
| Color-coded or other types of prioritization to report results | 206 (70%) | 11 | 79 |
| Age/gender related rules | 240 (81%) | 9 | 47 |
| Westgard rules | 199 (67%) | 21 | 76 |
| Generation of statistics reports | 231 (78%) | 18 | 47 |
| Delta Checks | 153 (52%) | 25 | 118 |
| Serum Index Checks | 144 (49%) | 25 | 127 |
| QC Monitoring | 227 (77%) | 19 | 50 |
| Turnaround time monitoring | 244 (82%) | 14 | 38 |
| Sample archiving | 172 (58%) | 22 | 102 |

#### Commercial laboratory

|  | Currently use | Plan to use in the next 12 months | Do not use |
| --- | --- | --- | --- |
| Tracking costs per tests | 106 (48%) | 19 | 98 |
| Color-coded or other types of prioritization to report results | 146 (65%) | 9 | 68 |
| Age/gender related rules | 185 (83%) | 7 | 31 |
| Westgard rules | 173 (78%) | 12 | 38 |
| Generation of statistics reports | 168 (75%) | 11 | 44 |
| Delta Checks | 151 (68%) | 14 | 58 |
| Serum Index Checks | 132 (59%) | 21 | 70 |
| QC Monitoring | 181 (81%) | 15 | 27 |
| Turnaround time monitoring | 173 (78%) | 12 | 38 |
| Sample archiving | 158 (71%) | 8 | 57 |

## Item 18 - Advanced IT functionalities

### Prompt

Do you use any of the advanced IT functionalities listed below (pick one for each of the following)?

- Audit trail (end to end traceability for reagents, controls and consumables)
- Demand (workload) & capacity alignment (i.e. instruments, staff)
- Reagent supply monitoring/ordering
- Pre Lab sample tracking (i.e. during shipment)
- Sample location tracking throughout the lab
- Information Dashboard to display KPI’s
- Real time turnaround time monitoring (i.e. Andon board)
- Messaging, Push Mail, Alerting (e.g. smart phone)
- Patient result monitoring using floating median/moving averages
- Display cell morphology images in the Data Manager (i.e. LIS)

### Results

#### Combined

|  | Currently use | Plan to use in the next 12 months | Do not use |
| --- | --- | --- | --- |
| Audit trail (end to end traceability for reagents, controls and consumables) | 385 (42%) | 96 | 439 |
| Demand (workload) & capacity alignment (i.e. instruments, staff) | 224 (24%) | 103 | 593 |
| Reagent supply monitoring/ordering | 454 (49%) | 134 | 332 |
| Pre Lab sample tracking (i.e. during shipment) | 252 (27%) | 124 | 544 |
| Sample location tracking throughout the lab | 336 (37%) | 85 | 499 |
| Information Dashboard to display KPI’s | 217 (24%) | 110 | 593 |
| Real time turnaround time monitoring (i.e. Andon board) | 316 (34%) | 87 | 517 |
| Messaging, Push Mail, Alerting (e.g. smart phone) | 314 (34%) | 77 | 529 |
| Patient result monitoring using floating median/moving averages | 190 (21%) | 103 | 627 |
| Display cell morphology images in the Data Manager (i.e. LIS) | 199 (22%) | 84 | 637 |

#### Government hospital laboratory

|  | Currently use | Plan to use in the next 12 months | Do not use |
| --- | --- | --- | --- |
| Audit trail (end to end traceability for reagents, controls and consumables) | 134 (33%) | 42 | 225 |
| Demand (workload) & capacity alignment (i.e. instruments, staff) | 79 (20%) | 48 | 274 |
| Reagent supply monitoring/ordering | 179 (45%) | 49 | 173 |
| Pre Lab sample tracking (i.e. during shipment) | 92 (23%) | 35 | 274 |
| Sample location tracking throughout the lab | 166 (41%) | 33 | 202 |
| Information Dashboard to display KPI’s | 76 (19%) | 46 | 279 |
| Real time turnaround time monitoring (i.e. Andon board) | 128 (32%) | 40 | 233 |
| Messaging, Push Mail, Alerting (e.g. smart phone) | 80 (20%) | 35 | 286 |
| Patient result monitoring using floating median/moving averages | 79 (20%) | 49 | 273 |
| Display cell morphology images in the Data Manager (i.e. LIS) | 88 (22%) | 38 | 275 |

#### Private hospital laboratory

|  | Currently use | Plan to use in the next 12 months | Do not use |
| --- | --- | --- | --- |
| Audit trail (end to end traceability for reagents, controls and consumables) | 138 (47%) | 29 | 129 |
| Demand (workload) & capacity alignment (i.e. instruments, staff) | 67 (23%) | 25 | 204 |
| Reagent supply monitoring/ordering | 138 (47%) | 64 | 94 |
| Pre Lab sample tracking (i.e. during shipment) | 74 (25%) | 65 | 157 |
| Sample location tracking throughout the lab | 81 (27%) | 34 | 181 |
| Information Dashboard to display KPI’s | 84 (28%) | 37 | 175 |
| Real time turnaround time monitoring (i.e. Andon board) | 122 (41%) | 28 | 146 |
| Messaging, Push Mail, Alerting (e.g. smart phone) | 125 (42%) | 28 | 143 |
| Patient result monitoring using floating median/moving averages | 45 (15%) | 34 | 217 |
| Display cell morphology images in the Data Manager (i.e. LIS) | 52 (18%) | 29 | 215 |

#### Commercial laboratory

|  | Currently use | Plan to use in the next 12 months | Do not use |
| --- | --- | --- | --- |
| Audit trail (end to end traceability for reagents, controls and consumables) | 113 (51%) | 25 | 85 |
| Demand (workload) & capacity alignment (i.e. instruments, staff) | 78 (35%) | 30 | 115 |
| Reagent supply monitoring/ordering | 137 (61%) | 21 | 65 |
| Pre Lab sample tracking (i.e. during shipment) | 86 (39%) | 24 | 113 |
| Sample location tracking throughout the lab | 89 (40%) | 18 | 116 |
| Information Dashboard to display KPI’s | 57 (26%) | 27 | 139 |
| Real time turnaround time monitoring (i.e. Andon board) | 66 (30%) | 19 | 138 |
| Messaging, Push Mail, Alerting (e.g. smart phone) | 109 (49%) | 14 | 100 |
| Patient result monitoring using floating median/moving averages | 66 (30%) | 20 | 137 |
| Display cell morphology images in the Data Manager (i.e. LIS) | 59 (26%) | 17 | 147 |

## Item 19 - Automation

### Prompt

Which workflow steps are integrated into your automation solution (pick one for each of the following)?

- Bulk loading
- Direct pneumatic tube loading
- Rack loading
- Sample quality check (HIL)
- Sample Volume check
- Vortexing
- Centrifugation
- Sorting into instrument compatible racks
- Decapping
- Aliquotting
- Tube labelling
- Sample transportation via track
- Vertical sample transportation
- Recapping
- Short term storage (i.e. buffer)
- Mid term storage (i.e. cooled archiving)
- Long term storage (i.e. Biobanking)
- Reflex or add-on testing
- Sample disposal

### Results

#### Combined

|  | Currently integrated | Plan to integrate in the next 12 months | Not integrated |
| --- | --- | --- | --- |
| Bulk loading | 165 (18%) | 44 | 711 |
| Direct pneumatic tube loading | 117 (13%) | 44 | 759 |
| Rack loading | 352 (38%) | 46 | 522 |
| Sample quality check (HIL) | 381 (41%) | 58 | 480 |
| Sample Volume check | 358 (39%) | 45 | 517 |
| Vortexing | 118 (13%) | 40 | 762 |
| Centrifugation | 273 (30%) | 52 | 595 |
| Sorting into instrument compatible racks | 255 (28%) | 48 | 617 |
| Decapping | 300 (33%) | 47 | 573 |
| Aliquotting | 228 (25%) | 48 | 644 |
| Tube labelling | 218 (24%) | 46 | 656 |
| sample transportation via track | 250 (27%) | 56 | 614 |
| vertical sample transportation | 99 (11%) | 38 | 782 |
| Recapping | 225 (24%) | 54 | 641 |
| Short term storage (i.e. buffer) | 217 (24%) | 58 | 645 |
| Mid term storage (i.e. cooled archiving) | 203 (22%) | 50 | 667 |
| Long term storage (i.e. Biobanking) | 92 (10%) | 38 | 790 |
| Reflex or add-on testing | 293 (32%) | 61 | 566 |
| Sample disposal | 197 (21%) | 46 | 677 |

#### Government hospital laboratory

|  | Currently integrated | Plan to integrate in the next 12 months | Not integrated |
| --- | --- | --- | --- |
| Bulk loading | 85 (21%) | 20 | 296 |
| Direct pneumatic tube loading | 53 (13%) | 25 | 323 |
| Rack loading | 192 (48%) | 21 | 188 |
| Sample quality check (HIL) | 191 (48%) | 20 | 190 |
| Sample Volume check | 177 (44%) | 22 | 202 |
| Vortexing | 56 (14%) | 15 | 330 |
| Centrifugation | 151 (38%) | 23 | 227 |
| Sorting into instrument compatible racks | 143 (36%) | 19 | 239 |
| Decapping | 181 (45%) | 17 | 203 |
| Aliquotting | 132 (33%) | 19 | 250 |
| Tube labelling | 129 (32%) | 19 | 253 |
| sample transportation via track | 157 (39%) | 25 | 219 |
| vertical sample transportation | 52 (13%) | 13 | 335 |
| Recapping | 139 (35%) | 22 | 240 |
| Short term storage (i.e. buffer) | 119 (30%) | 29 | 253 |
| Mid term storage (i.e. cooled archiving) | 114 (28%) | 21 | 266 |
| Long term storage (i.e. Biobanking) | 53 (13%) | 12 | 336 |
| Reflex or add-on testing | 155 (39%) | 27 | 219 |
| Sample disposal | 114 (28%) | 18 | 269 |

#### Private hospital laboratory

|  | Currently integrated | Plan to integrate in the next 12 months | Not integrated |
| --- | --- | --- | --- |
| Bulk loading | 43 (15%) | 15 | 238 |
| Direct pneumatic tube loading | 44 (15%) | 13 | 239 |
| Rack loading | 85 (29%) | 18 | 193 |
| Sample quality check (HIL) | 101 (34%) | 23 | 172 |
| Sample Volume check | 91 (31%) | 16 | 189 |
| Vortexing | 31 (10%) | 16 | 249 |
| Centrifugation | 64 (22%) | 19 | 213 |
| Sorting into instrument compatible racks | 63 (21%) | 18 | 215 |
| Decapping | 69 (23%) | 19 | 208 |
| Aliquotting | 54 (18%) | 19 | 223 |
| Tube labelling | 51 (17%) | 18 | 227 |
| sample transportation via track | 58 (20%) | 21 | 217 |
| vertical sample transportation | 29 (10%) | 17 | 250 |
| Recapping | 50 (17%) | 19 | 227 |
| Short term storage (i.e. buffer) | 50 (17%) | 19 | 227 |
| Mid term storage (i.e. cooled archiving) | 48 (16%) | 17 | 231 |
| Long term storage (i.e. Biobanking) | 18 (6%) | 15 | 263 |
| Reflex or add-on testing | 70 (24%) | 25 | 201 |
| Sample disposal | 44 (15%) | 20 | 232 |

#### Commercial laboratory

|  | Currently integrated | Plan to integrate in the next 12 months | Not integrated |
| --- | --- | --- | --- |
| Bulk loading | 37 (17%) | 9 | 177 |
| Direct pneumatic tube loading | 20 (9%) | 6 | 197 |
| Rack loading | 75 (34%) | 7 | 141 |
| Sample quality check (HIL) | 89 (40%) | 15 | 118 |
| Sample Volume check | 90 (40%) | 7 | 126 |
| Vortexing | 31 (14%) | 9 | 183 |
| Centrifugation | 58 (26%) | 10 | 155 |
| Sorting into instrument compatible racks | 49 (22%) | 11 | 163 |
| Decapping | 50 (22%) | 11 | 162 |
| Aliquotting | 42 (19%) | 10 | 171 |
| Tube labelling | 38 (17%) | 9 | 176 |
| sample transportation via track | 35 (16%) | 10 | 178 |
| vertical sample transportation | 18 (8%) | 8 | 197 |
| Recapping | 36 (16%) | 13 | 174 |
| Short term storage (i.e. buffer) | 48 (22%) | 10 | 165 |
| Mid term storage (i.e. cooled archiving) | 41 (18%) | 12 | 170 |
| Long term storage (i.e. Biobanking) | 21 (9%) | 11 | 191 |
| Reflex or add-on testing | 68 (30%) | 9 | 146 |
| Sample disposal | 39 (17%) | 8 | 176 |

## Item 20 - Analyzers connected to track

### Prompt

Which analysers are connected to a track to automate sample transportation (pick one for each of the following)?

- Integrated CC/IA
- Clinical Chemistry
- Immunoassays incl. serology (i.e. Hormones-, Tumor-, Cardiac- Markers, SARS-CoV-2)
- Haematology
- Coagulation
- Urinalysis
- Routine Molecular Diagnostics (i.e. HPV, HIV, HBV, CT/NG, SARS-CoV-2)

### Results

#### Combined

|  | Track connected analysers | Plan to connect in next 12 months | Stand alone analysers | N/A |
| --- | --- | --- | --- | --- |
| Integrated CC/IA | 187 (20%) | 58 | 186 | 489 |
| Clinical Chemistry | 239 (26%) | 46 | 218 | 417 |
| Immunoassays incl. serology (i.e. Hormones-, Tumor-, Cardiac- Markers, SARS-CoV-2) | 225 (24%) | 48 | 217 | 430 |
| Haematology | 81 (9%) | 55 | 336 | 448 |
| Coagulation | 68 (7%) | 40 | 332 | 480 |
| Urinalysis | 46 (5%) | 24 | 353 | 497 |
| Routine Molecular Diagnostics (i.e. HPV, HIV, HBV, CT/NG, SARS-CoV-2) | 22 (2%) | 17 | 262 | 619 |

#### Government hospital laboratory

|  | Track connected analysers | Plan to connect in next 12 months | Stand alone analysers | N/A |
| --- | --- | --- | --- | --- |
| Integrated CC/IA | 101 (25%) | 28 | 71 | 201 |
| Clinical Chemistry | 148 (37%) | 20 | 69 | 164 |
| Immunoassays incl. serology (i.e. Hormones-, Tumor-, Cardiac- Markers, SARS-CoV-2) | 138 (34%) | 23 | 77 | 163 |
| Haematology | 51 (13%) | 26 | 132 | 192 |
| Coagulation | 53 (13%) | 20 | 127 | 201 |
| Urinalysis | 22 (5%) | 6 | 159 | 214 |
| Routine Molecular Diagnostics (i.e. HPV, HIV, HBV, CT/NG, SARS-CoV-2) | 14 (3%) | 3 | 124 | 260 |

#### Private hospital laboratory

|  | Track connected analysers | Plan to connect in next 12 months | Stand alone analysers | N/A |
| --- | --- | --- | --- | --- |
| Integrated CC/IA | 53 (18%) | 16 | 65 | 162 |
| Clinical Chemistry | 60 (20%) | 14 | 89 | 133 |
| Immunoassays incl. serology (i.e. Hormones-, Tumor-, Cardiac- Markers, SARS-CoV-2) | 56 (19%) | 14 | 84 | 142 |
| Haematology | 19 (6%) | 17 | 122 | 138 |
| Coagulation | 11 (4%) | 13 | 126 | 146 |
| Urinalysis | 10 (3%) | 9 | 134 | 143 |
| Routine Molecular Diagnostics (i.e. HPV, HIV, HBV, CT/NG, SARS-CoV-2) | 7 (2%) | 8 | 87 | 194 |

#### Commercial laboratory

|  | Track connected analysers | Plan to connect in next 12 months | Stand alone analysers | N/A |
| --- | --- | --- | --- | --- |
| Integrated CC/IA | 33 (15%) | 14 | 50 | 126 |
| Clinical Chemistry | 31 (14%) | 12 | 60 | 120 |
| Immunoassays incl. serology (i.e. Hormones-, Tumor-, Cardiac- Markers, SARS-CoV-2) | 31 (14%) | 11 | 56 | 125 |
| Haematology | 11 (5%) | 12 | 82 | 118 |
| Coagulation | 4 (2%) | 7 | 79 | 133 |
| Urinalysis | 14 (6%) | 9 | 60 | 140 |
| Routine Molecular Diagnostics (i.e. HPV, HIV, HBV, CT/NG, SARS-CoV-2) | 1 (0%) | 6 | 51 | 165 |

# Items 21-30

## Item 21 - Main manufacturer

To keep respondents anonymous, this item has not been analyzed (cf. Introduction).

## Item 22 - Number of analyzers / modules

To keep respondents anonymous, this item has not been analyzed (cf. Introduction).

## Item 23 - Influence on healthcare system

### Prompt

Does your laboratory monitor its impact on any of the Healthcare System KPI’s listed below (pick one for each of the following):

- A&E (Emergency Department) waiting times
- Delays of Surgical Procedures
- Discharge delays; Ward Length of Stay
- Reducing readmissions
- Speeding up diagnosis and treatments
- Patients falling into care gaps
- Identifying patients at risk; prevention and risk stratification
- Primary Care initiatives with GP’s
- Geographic spread of infectious diseases
- Mortality rates

### Results

#### Combined

|  | Monitor currently | Plan to monitor in next 12 months | Do not monitor |
| --- | --- | --- | --- |
| A&E (Emergency Department) waiting times | 278 (30%) | 39 | 603 |
| Delays of Surgical Procedures | 154 (17%) | 29 | 737 |
| Discharge delays; Ward Length of Stay | 130 (14%) | 35 | 755 |
| Reducing readmissions | 104 (11%) | 40 | 776 |
| Speeding up diagnosis and treatments | 173 (19%) | 45 | 702 |
| Patients falling into care gaps | 96 (10%) | 31 | 793 |
| Identifying patients at risk; prevention and risk stratification | 189 (21%) | 54 | 677 |
| Primary Care initiatives with GP’s | 153 (17%) | 44 | 723 |
| Geographic spread of infectious diseases | 155 (17%) | 42 | 723 |
| Mortality rates | 129 (14%) | 30 | 761 |

#### Government hospital laboratory

|  | Monitor currently | Plan to monitor in next 12 months | Do not monitor |
| --- | --- | --- | --- |
| A&E (Emergency Department) waiting times | 106 (26%) | 20 | 275 |
| Delays of Surgical Procedures | 68 (17%) | 11 | 322 |
| Discharge delays; Ward Length of Stay | 57 (14%) | 10 | 334 |
| Reducing readmissions | 48 (12%) | 17 | 336 |
| Speeding up diagnosis and treatments | 75 (19%) | 19 | 307 |
| Patients falling into care gaps | 49 (12%) | 10 | 342 |
| Identifying patients at risk; prevention and risk stratification | 92 (23%) | 16 | 293 |
| Primary Care initiatives with GP’s | 69 (17%) | 19 | 313 |
| Geographic spread of infectious diseases | 69 (17%) | 17 | 315 |
| Mortality rates | 62 (15%) | 12 | 327 |

#### Private hospital laboratory

|  | Monitor currently | Plan to monitor in next 12 months | Do not monitor |
| --- | --- | --- | --- |
| A&E (Emergency Department) waiting times | 137 (46%) | 14 | 145 |
| Delays of Surgical Procedures | 68 (23%) | 13 | 215 |
| Discharge delays; Ward Length of Stay | 59 (20%) | 21 | 216 |
| Reducing readmissions | 50 (17%) | 19 | 227 |
| Speeding up diagnosis and treatments | 64 (22%) | 20 | 212 |
| Patients falling into care gaps | 40 (14%) | 17 | 239 |
| Identifying patients at risk; prevention and risk stratification | 61 (21%) | 28 | 207 |
| Primary Care initiatives with GP’s | 51 (17%) | 21 | 224 |
| Geographic spread of infectious diseases | 50 (17%) | 19 | 227 |
| Mortality rates | 61 (21%) | 14 | 221 |

#### Commercial laboratory

|  | Monitor currently | Plan to monitor in next 12 months | Do not monitor |
| --- | --- | --- | --- |
| A&E (Emergency Department) waiting times | 35 (16%) | 5 | 183 |
| Delays of Surgical Procedures | 18 (8%) | 5 | 200 |
| Discharge delays; Ward Length of Stay | 14 (6%) | 4 | 205 |
| Reducing readmissions | 6 (3%) | 4 | 213 |
| Speeding up diagnosis and treatments | 34 (15%) | 6 | 183 |
| Patients falling into care gaps | 7 (3%) | 4 | 212 |
| Identifying patients at risk; prevention and risk stratification | 36 (16%) | 10 | 177 |
| Primary Care initiatives with GP’s | 33 (15%) | 4 | 186 |
| Geographic spread of infectious diseases | 36 (16%) | 6 | 181 |
| Mortality rates | 6 (3%) | 4 | 213 |

## Item 24 - Brain injury management

### Prompt

Does your laboratory play a role in the management of patients with suspected minor traumatic brain injury (pick one)?

- Currently do
- Plan to do in the next 12 months
- Don’t do

### Results

|  | Currently do | Plan to do in the next 12 months | Don’t do |
| --- | --- | --- | --- |
| Combined | 155 (17%) | 89 | 676 |
| Government hospital laboratory | 83 (21%) | 48 | 270 |
| Private hospital laboratory | 55 (19%) | 28 | 213 |
| Commercial laboratory | 17 (8%) | 13 | 193 |

## Item 25 - Services to physicians

### Prompt

Which of the following services does your laboratory provide to physicians in addition to results (pick one for each of the following)?

- Interpretation of results
- Reflexive test suggestions
- Proactive consultation on complex patient cases
- Diagnostic pathway guidance
- Optimisation of adherence to diagnostic guidelines
- Therapeutic recommendation
- Real time decision support using clinical algorithms
- Guidance based on historical patient results
- Guidance on over- and under- utilization of testing
- Continuous education events

### Results

#### Combined

|  | Provide currently | Plan to provide in next 12 months | Do not provide |
| --- | --- | --- | --- |
| Interpretation of results | 759 (82%) | 21 | 140 |
| Reflexive test suggestions | 606 (66%) | 30 | 283 |
| Proactive consultation on complex patient cases | 536 (58%) | 43 | 341 |
| Diagnostic pathway guidance | 447 (49%) | 53 | 420 |
| Optimisation of adherence to diagnostic guidelines | 359 (39%) | 62 | 499 |
| Therapeutic recommendation | 281 (31%) | 36 | 603 |
| Real time decision support using clinical algorithms | 234 (25%) | 67 | 619 |
| Guidance based on historical patient results | 464 (51%) | 43 | 411 |
| Guidance on over- and under-utilization of testing | 361 (39%) | 63 | 495 |
| Continuous education events | 498 (54%) | 63 | 358 |

#### Government hospital laboratory

|  | Provide currently | Plan to provide in next 12 months | Do not provide |
| --- | --- | --- | --- |
| Interpretation of results | 319 (80%) | 14 | 68 |
| Reflexive test suggestions | 259 (65%) | 18 | 123 |
| Proactive consultation on complex patient cases | 251 (63%) | 25 | 125 |
| Diagnostic pathway guidance | 196 (49%) | 29 | 176 |
| Optimisation of adherence to diagnostic guidelines | 159 (40%) | 31 | 211 |
| Therapeutic recommendation | 121 (30%) | 18 | 262 |
| Real time decision support using clinical algorithms | 98 (24%) | 33 | 270 |
| Guidance based on historical patient results | 214 (54%) | 20 | 166 |
| Guidance on over- and under-utilization of testing | 185 (46%) | 37 | 179 |
| Continuous education events | 212 (53%) | 35 | 153 |

#### Private hospital laboratory

|  | Provide currently | Plan to provide in next 12 months | Do not provide |
| --- | --- | --- | --- |
| Interpretation of results | 243 (82%) | 5 | 48 |
| Reflexive test suggestions | 188 (64%) | 8 | 100 |
| Proactive consultation on complex patient cases | 161 (54%) | 7 | 128 |
| Diagnostic pathway guidance | 133 (45%) | 12 | 151 |
| Optimisation of adherence to diagnostic guidelines | 105 (35%) | 18 | 173 |
| Therapeutic recommendation | 74 (25%) | 9 | 213 |
| Real time decision support using clinical algorithms | 60 (20%) | 23 | 213 |
| Guidance based on historical patient results | 122 (41%) | 13 | 160 |
| Guidance on over- and under-utilization of testing | 97 (33%) | 16 | 182 |
| Continuous education events | 149 (50%) | 18 | 129 |

#### Commercial laboratory

|  | Provide currently | Plan to provide in next 12 months | Do not provide |
| --- | --- | --- | --- |
| Interpretation of results | 197 (88%) | 2 | 24 |
| Reflexive test suggestions | 159 (71%) | 4 | 60 |
| Proactive consultation on complex patient cases | 124 (56%) | 11 | 88 |
| Diagnostic pathway guidance | 118 (53%) | 12 | 93 |
| Optimisation of adherence to diagnostic guidelines | 95 (43%) | 13 | 115 |
| Therapeutic recommendation | 86 (39%) | 9 | 128 |
| Real time decision support using clinical algorithms | 76 (34%) | 11 | 136 |
| Guidance based on historical patient results | 128 (57%) | 10 | 85 |
| Guidance on over- and under-utilization of testing | 79 (35%) | 10 | 134 |
| Continuous education events | 137 (61%) | 10 | 76 |

## Item 26 - Physician satisfaction

### Prompt

How satisfied do you think your requesting physicians are with the services offered by your laboratory (pick one)?

- Satisfied
- Neutral
- Dissatisfied

### Results

|  | Satisfied | Neutral | Dissatisfied |
| --- | --- | --- | --- |
| Combined | 738 (80%) | 178 | 4 |
| Government hospital laboratory | 315 (79%) | 84 | 2 |
| Private hospital laboratory | 218 (74%) | 78 | 0 |
| Commercial laboratory | 205 (92%) | 16 | 2 |

## Item 27 - Communication channels

### Prompt

What channels of communication do you use to promote your services to patients and physicians (pick one for each of the following)?

- Email
- Social media
- Website
- Newsletter
- Print media
- Events

### Results

#### Combined

|  | Currently use | Plan to use in the next 12 months | Do not use |
| --- | --- | --- | --- |
| Email | 656 (71%) | 11 | 253 |
| Social media | 451 (49%) | 29 | 440 |
| Website | 609 (66%) | 31 | 280 |
| Newsletter | 423 (46%) | 31 | 466 |
| Print media | 407 (44%) | 24 | 489 |
| Events | 489 (53%) | 40 | 391 |

#### Government hospital laboratory

|  | Currently use | Plan to use in the next 12 months | Do not use |
| --- | --- | --- | --- |
| Email | 255 (64%) | 3 | 143 |
| Social media | 104 (26%) | 13 | 284 |
| Website | 214 (53%) | 17 | 170 |
| Newsletter | 131 (33%) | 13 | 257 |
| Print media | 118 (29%) | 10 | 273 |
| Events | 180 (45%) | 16 | 205 |

#### Private hospital laboratory

|  | Currently use | Plan to use in the next 12 months | Do not use |
| --- | --- | --- | --- |
| Email | 233 (79%) | 4 | 59 |
| Social media | 182 (61%) | 9 | 105 |
| Website | 209 (71%) | 9 | 78 |
| Newsletter | 165 (56%) | 11 | 120 |
| Print media | 162 (55%) | 10 | 124 |
| Events | 177 (60%) | 14 | 105 |

#### Commercial laboratory

|  | Currently use | Plan to use in the next 12 months | Do not use |
| --- | --- | --- | --- |
| Email | 168 (75%) | 4 | 51 |
| Social media | 165 (74%) | 7 | 51 |
| Website | 186 (83%) | 5 | 32 |
| Newsletter | 127 (57%) | 7 | 89 |
| Print media | 127 (57%) | 4 | 92 |
| Events | 132 (59%) | 10 | 81 |

## Item 28 - Outreach strategy

### Prompt

Do you make use of clinical lab data as suggested below (pick one for each of the following)?

- Disease surveillance and/or outbreak management
- Partnership with public or private payers to leverage lab data for risk management
- Outcome based pricing or reimbursement schemes that reward labs for value creation
- Preventative health and wellness programs
- Contribution to Population Health Initiatives
- Referral of patients to others services or specialists

### Results

#### Combined

|  | Currently use | Plan to use in the next 12 months | Do not use |
| --- | --- | --- | --- |
| Disease surveillance and/or outbreak management | 412 (45%) | 36 | 472 |
| Partnership with public or private payers to leverage lab data for risk management | 191 (21%) | 42 | 687 |
| Outcome based pricing or reimbursement schemes that reward labs for value creation | 119 (13%) | 39 | 762 |
| Preventative health and wellness programs | 356 (39%) | 54 | 510 |
| Contribution to Population Health Initiatives | 313 (34%) | 48 | 559 |
| Referral of patients to others services or specialists | 401 (44%) | 26 | 493 |

#### Government hospital laboratory

|  | Currently use | Plan to use in the next 12 months | Do not use |
| --- | --- | --- | --- |
| Disease surveillance and/or outbreak management | 181 (45%) | 19 | 201 |
| Partnership with public or private payers to leverage lab data for risk management | 90 (22%) | 19 | 292 |
| Outcome based pricing or reimbursement schemes that reward labs for value creation | 48 (12%) | 21 | 332 |
| Preventative health and wellness programs | 124 (31%) | 28 | 249 |
| Contribution to Population Health Initiatives | 134 (33%) | 27 | 240 |
| Referral of patients to others services or specialists | 167 (42%) | 13 | 221 |

#### Private hospital laboratory

|  | Currently use | Plan to use in the next 12 months | Do not use |
| --- | --- | --- | --- |
| Disease surveillance and/or outbreak management | 152 (51%) | 13 | 131 |
| Partnership with public or private payers to leverage lab data for risk management | 51 (17%) | 15 | 230 |
| Outcome based pricing or reimbursement schemes that reward labs for value creation | 44 (15%) | 14 | 238 |
| Preventative health and wellness programs | 140 (47%) | 17 | 139 |
| Contribution to Population Health Initiatives | 90 (30%) | 16 | 190 |
| Referral of patients to others services or specialists | 151 (51%) | 9 | 136 |

### Commercial laboratory

|  | Currently use | Plan to use in the next 12 months | Do not use |
| --- | --- | --- | --- |
| Disease surveillance and/or outbreak management | 79 (35%) | 4 | 140 |
| Partnership with public or private payers to leverage lab data for risk management | 50 (22%) | 8 | 165 |
| Outcome based pricing or reimbursement schemes that reward labs for value creation | 27 (12%) | 4 | 192 |
| Preventative health and wellness programs | 92 (41%) | 9 | 122 |
| Contribution to Population Health Initiatives | 89 (40%) | 5 | 129 |
| Referral of patients to others services or specialists | 83 (37%) | 4 | 136 |

## Item 29 - Clinical chemistry tubes

### Prompt

How many primary **TUBES** do you process per day in Clinical Chemistry?

{Slider from 0 to 20000}

### Results

|  | n | Max. | 3rd Qu. | Mean | SD | Median | IQR | 1st Qu. | Min. |
| --- | --- | --- | --- | --- | --- | --- | --- | --- | --- |
| Combined | 920 | 20000 | 1000 | 1077 | 2310 | 400 | 800 | 200 | 0 |
| Government hospital laboratory | 401 | 20000 | 1293 | 1241 | 2282 | 600 | 993 | 300 | 0 |
| Private hospital laboratory | 296 | 20000 | 600 | 709 | 1772 | 300 | 450 | 150 | 8 |
| Commercial laboratory | 223 | 20000 | 1200 | 1269 | 2874 | 300 | 1100 | 100 | 0 |

## Item 30 - Clinical chemistry tests

### Prompt

How many Clinical Chemistry **TESTS** do you process per day?

{Slider from 0 to 300000}

### Results

|  | n | Max. | 3rd Qu. | Mean | SD | Median | IQR | 1st Qu. | Min. |
| --- | --- | --- | --- | --- | --- | --- | --- | --- | --- |
| Combined | 920 | 300000 | 7150 | 9593 | 30276 | 2620 | 6400 | 750 | 0 |
| Government hospital laboratory | 401 | 300000 | 10006 | 11682 | 29065 | 4500 | 8093 | 1913 | 0 |
| Private hospital laboratory | 296 | 232290 | 4000 | 5483 | 20366 | 1500 | 3500 | 500 | 15 |
| Commercial laboratory | 223 | 300000 | 5000 | 11294 | 41086 | 1654 | 4500 | 500 | 0 |

# Items 31-40

## Item 31 - IA and serology tubes

### Prompt

How many primary **TUBES** do you process per day in Immunoassays including serology?

{Slider from 0 to 20000}

### Results

|  | n | Max. | 3rd Qu. | Mean | SD | Median | IQR | 1st Qu. | Min. |
| --- | --- | --- | --- | --- | --- | --- | --- | --- | --- |
| Combined | 920 | 20000 | 400 | 567 | 1789 | 200 | 330 | 70 | 0 |
| Government hospital laboratory | 401 | 20000 | 500 | 517 | 1507 | 250 | 400 | 100 | 0 |
| Private hospital laboratory | 296 | 10000 | 300 | 313 | 851 | 120 | 250 | 50 | 0 |
| Commercial laboratory | 223 | 20000 | 700 | 995 | 2813 | 195 | 650 | 50 | 0 |

## Item 32 - IA and serology tests

### Prompt

How many Immunoassay **TESTS** do you process per day including serology?

{Slider from 0 to 300000}

### Results

|  | n | Max. | 3rd Qu. | Mean | SD | Median | IQR | 1st Qu. | Min. |
| --- | --- | --- | --- | --- | --- | --- | --- | --- | --- |
| Combined | 920 | 300000 | 1500 | 3807 | 21527 | 500 | 1305 | 195 | 0 |
| Government hospital laboratory | 401 | 300000 | 1537 | 3564 | 19256 | 720 | 1287 | 250 | 0 |
| Private hospital laboratory | 296 | 86884 | 1000 | 1537 | 6405 | 300 | 881 | 119 | 0 |
| Commercial laboratory | 223 | 300000 | 2500 | 7256 | 34306 | 600 | 2352 | 148 | 0 |

## Item 33 - Haematology tubes

### Prompt

How many primary **TUBES** do you process per day in Haematology?

{Slider from 0 to 20000}

### Results

|  | n | Max. | 3rd Qu. | Mean | SD | Median | IQR | 1st Qu. | Min. |
| --- | --- | --- | --- | --- | --- | --- | --- | --- | --- |
| Combined | 920 | 20000 | 500 | 562 | 1546 | 250 | 400 | 100 | 0 |
| Government hospital laboratory | 401 | 20000 | 700 | 672 | 1598 | 380 | 535 | 165 | 0 |
| Private hospital laboratory | 296 | 2943 | 361 | 313 | 386 | 200 | 261 | 100 | 0 |
| Commercial laboratory | 223 | 20000 | 448 | 692 | 2231 | 150 | 398 | 50 | 0 |

## Item 34 - Haematology tests

### Prompt

How many Haematology **CBC TESTS** (not parameters) do you process per day?

{Slider from 0 to 300000}

### Results

|  | n | Max. | 3rd Qu. | Mean | SD | Median | IQR | 1st Qu. | Min. |
| --- | --- | --- | --- | --- | --- | --- | --- | --- | --- |
| Combined | 920 | 300000 | 600 | 1370 | 11209 | 274 | 500 | 100 | 0 |
| Government hospital laboratory | 401 | 300000 | 800 | 1838 | 15398 | 400 | 609 | 191 | 0 |
| Private hospital laboratory | 296 | 83688 | 400 | 724 | 4907 | 200 | 300 | 100 | 0 |
| Commercial laboratory | 223 | 95318 | 530 | 1386 | 7744 | 170 | 470 | 60 | 0 |

## Item 35 - Coagulation tubes

### Prompt

How many primary **TUBES** do you process per day in Coagulation?

{Slider from 0 to 20000}

### Results

|  | n | Max. | 3rd Qu. | Mean | SD | Median | IQR | 1st Qu. | Min. |
| --- | --- | --- | --- | --- | --- | --- | --- | --- | --- |
| Combined | 920 | 20000 | 150 | 226 | 1328 | 50 | 132 | 18 | 0 |
| Government hospital laboratory | 401 | 20000 | 250 | 327 | 1651 | 100 | 200 | 50 | 0 |
| Private hospital laboratory | 296 | 2000 | 80 | 78 | 160 | 30 | 67 | 13 | 0 |
| Commercial laboratory | 223 | 20000 | 96 | 243 | 1518 | 35 | 86 | 10 | 0 |

## Item 36 - Coagulation tests

### Prompt

How many Coagulation **TESTS** do you process per day?

{Slider from 0 to 300000}

### Results

|  | n | Max. | 3rd Qu. | Mean | SD | Median | IQR | 1st Qu. | Min. |
| --- | --- | --- | --- | --- | --- | --- | --- | --- | --- |
| Combined | 920 | 300000 | 300 | 1054 | 11244 | 100 | 280 | 20 | 0 |
| Government hospital laboratory | 401 | 300000 | 600 | 1474 | 15221 | 200 | 533 | 67 | 0 |
| Private hospital laboratory | 296 | 22570 | 150 | 242 | 1361 | 60 | 130 | 20 | 0 |
| Commercial laboratory | 223 | 101338 | 150 | 1375 | 10105 | 50 | 135 | 15 | 0 |

## Item 37 - Urinalysis tubes

### Prompt

How many primary **TUBES/VIALS** do you process per day in Urinalysis?

{Slider from 0 to 20000}

### Results

|  | n | Max. | 3rd Qu. | Mean | SD | Median | IQR | 1st Qu. | Min. |
| --- | --- | --- | --- | --- | --- | --- | --- | --- | --- |
| Combined | 920 | 20000 | 200 | 286 | 1365 | 90 | 170 | 30 | 0 |
| Government hospital laboratory | 401 | 20000 | 250 | 309 | 1376 | 100 | 200 | 50 | 0 |
| Private hospital laboratory | 296 | 2916 | 150 | 142 | 232 | 80 | 116 | 34 | 0 |
| Commercial laboratory | 223 | 20000 | 200 | 435 | 2044 | 50 | 180 | 20 | 0 |

## Item 38 - Urinalysis tests

### Prompt

How many Urinalysis **TESTS** do you process per day?

{Slider from 0 to 300000}

### Results

|  | n | Max. | 3rd Qu. | Mean | SD | Median | IQR | 1st Qu. | Min. |
| --- | --- | --- | --- | --- | --- | --- | --- | --- | --- |
| Combined | 920 | 300000 | 500 | 1469 | 13142 | 150 | 450 | 50 | 0 |
| Government hospital laboratory | 401 | 300000 | 600 | 1753 | 15437 | 200 | 532 | 68 | 0 |
| Private hospital laboratory | 296 | 14514 | 350 | 561 | 1540 | 100 | 310 | 40 | 0 |
| Commercial laboratory | 223 | 197580 | 305 | 2165 | 16756 | 80 | 275 | 30 | 0 |

## Item 39 - Routine molecular tubes

### Prompt

How many primary **TUBES/VIALS** do you process per day in Routine Molecular Diagnostics?

{Slider from 0 to 20000}

### Results

|  | n | Max. | 3rd Qu. | Mean | SD | Median | IQR | 1st Qu. | Min. |
| --- | --- | --- | --- | --- | --- | --- | --- | --- | --- |
| Combined | 920 | 20000 | 100 | 296 | 1584 | 15 | 100 | 0 | 0 |
| Government hospital laboratory | 401 | 20000 | 100 | 243 | 1432 | 15 | 100 | 0 | 0 |
| Private hospital laboratory | 296 | 6000 | 50 | 123 | 497 | 15 | 50 | 0 | 0 |
| Commercial laboratory | 223 | 20000 | 213 | 619 | 2492 | 10 | 213 | 0 | 0 |

## Item 40 - Routine molecular tests

### Prompt

How many Routine Molecular Diagnostic **TESTS** do you process per day?

{Slider from 0 to 300000}

### Results

|  | n | Max. | 3rd Qu. | Mean | SD | Median | IQR | 1st Qu. | Min. |
| --- | --- | --- | --- | --- | --- | --- | --- | --- | --- |
| Combined | 920 | 300000 | 114 | 1097 | 14316 | 17 | 114 | 0 | 0 |
| Government hospital laboratory | 401 | 300000 | 150 | 1070 | 15092 | 20 | 150 | 0 | 0 |
| Private hospital laboratory | 296 | 6591 | 60 | 159 | 635 | 15 | 60 | 0 | 0 |
| Commercial laboratory | 223 | 300000 | 275 | 2392 | 20847 | 12 | 275 | 0 | 0 |

# Items 41-44

## Item 41 - Full time equivalents (FTEs)

### Prompt

Please indicate the **total number of FTEs** working in your laboratory and indicate in which section of the lab they are working (**FTE = Full Time Equivalent** = the equivalent of one person working 40h/week):

- Reception (i.e. accessioning)
- Pre-analytical Workstations (incl. sorting, centrifuge, aliquots, etc.)
- Analytical Workstations
- Post-analytical workstations (sample archiving, result validation)

### Results

#### Combined

| Section | n | Max. | 3rd Qu. | Mean | SD | Median | IQR | 1st Qu. | Min. |
| --- | --- | --- | --- | --- | --- | --- | --- | --- | --- |
| Reception | 920 | 400 | 5 | 6.8 | 18.6 | 3 | 3 | 2 | 0 |
| Pre-analytical | 920 | 120 | 5 | 5.6 | 10.0 | 3 | 4 | 1 | 0 |
| Analytical | 920 | 600 | 17 | 15.5 | 28.6 | 9 | 11 | 6 | 0 |
| Post-analytical | 920 | 200 | 6 | 6.0 | 12.4 | 3 | 5 | 1 | 0 |

#### Government hospital laboratory

| Section | n | Max. | 3rd Qu. | Mean | SD | Median | IQR | 1st Qu. | Min. |
| --- | --- | --- | --- | --- | --- | --- | --- | --- | --- |
| Reception | 401 | 200 | 6 | 6.6 | 13.7 | 3 | 4 | 2 | 0 |
| Pre-analytical | 401 | 120 | 5 | 5.7 | 10.6 | 3 | 3 | 2 | 0 |
| Analytical | 401 | 600 | 20 | 17.7 | 34.4 | 11 | 14 | 6 | 0 |
| Post-analytical | 401 | 120 | 6 | 6.2 | 10.0 | 3 | 4 | 2 | 0 |

#### Private hospital laboratory

| Section | n | Max. | 3rd Qu. | Mean | SD | Median | IQR | 1st Qu. | Min. |
| --- | --- | --- | --- | --- | --- | --- | --- | --- | --- |
| Reception | 296 | 123 | 4 | 5.1 | 11.8 | 2 | 3 | 1 | 0 |
| Pre-analytical | 296 | 100 | 5 | 4.8 | 9.0 | 2 | 4 | 1 | 0 |
| Analytical | 296 | 100 | 14 | 12.0 | 11.5 | 8 | 8 | 6 | 1 |
| Post-analytical | 296 | 100 | 5 | 5.2 | 10.0 | 2 | 4 | 1 | 0 |

#### Commercial laboratory

| Section | n | Max. | 3rd Qu. | Mean | SD | Median | IQR | 1st Qu. | Min. |
| --- | --- | --- | --- | --- | --- | --- | --- | --- | --- |
| Reception | 223 | 400 | 6 | 9.5 | 30.1 | 4 | 4 | 2 | 0 |
| Pre-analytical | 223 | 80 | 6 | 6.4 | 9.9 | 3 | 4 | 2 | 0 |
| Analytical | 223 | 350 | 16 | 16.4 | 32.6 | 8 | 11 | 5 | 0 |
| Post-analytical | 223 | 200 | 5 | 6.6 | 18.1 | 3 | 3 | 2 | 0 |

## Item 42 - FTEs for analysis

### Prompt

Please indicate how many **FTEs** mentioned in the **analytical workstations** above are in charge to operate the analytical instruments (Your total input should not exceed the amount indicated in analytical workstations in the previous question):

- Clinical Chemistry
- Immunoassays incl. serology (i.e. Hormones-, Tumor-, Cardiac- Markers, SARS-CoV-2)
- Haematology
- Coagulation
- Urinalysis
- Routine
- Molecular Diagnostics (i.e. HPV, HIV, HBV, CT/NG, SARS-CoV-2)

### Results

#### Combined

| Specialty | n | Max. | 3rd Qu. | Mean | SD | Median | IQR | 1st Qu. | Min. |
| --- | --- | --- | --- | --- | --- | --- | --- | --- | --- |
| Clinical chemistry | 908 | 113 | 5 | 4.1 | 6.9 | 2 | 4 | 1 | 0 |
| Immunoassays incl. serology | 879 | 40 | 4 | 3.1 | 3.5 | 2 | 3 | 1 | 0 |
| Haematology | 870 | 90 | 3 | 3.3 | 6.2 | 2 | 2 | 1 | 0 |
| Coagulation | 816 | 60 | 2 | 1.9 | 3.1 | 1 | 1 | 1 | 0 |
| Urinalysis | 830 | 80 | 2 | 2.2 | 4.6 | 1 | 1 | 1 | 0 |
| Routine molecular diagnostics | 575 | 70 | 3 | 3.0 | 5.2 | 2 | 2 | 1 | 0 |

#### Government hospital laboratory

| Specialty | n | Max. | 3rd Qu. | Mean | SD | Median | IQR | 1st Qu. | Min. |
| --- | --- | --- | --- | --- | --- | --- | --- | --- | --- |
| Clinical chemistry | 393 | 113 | 5 | 4.9 | 7.7 | 3 | 3 | 2 | 0 |
| Immunoassays incl. serology | 381 | 40 | 4 | 3.5 | 3.8 | 2 | 3 | 1 | 0 |
| Haematology | 362 | 87 | 4 | 3.9 | 6.4 | 2 | 3 | 1 | 0 |
| Coagulation | 339 | 60 | 2 | 2.2 | 3.9 | 1 | 1 | 1 | 0 |
| Urinalysis | 355 | 50 | 2 | 2.4 | 3.9 | 1 | 1 | 1 | 0 |
| Routine molecular diagnostics | 241 | 24 | 3 | 2.7 | 3.1 | 2 | 2 | 1 | 0 |

#### Private hospital laboratory

| Specialty | n | Max. | 3rd Qu. | Mean | SD | Median | IQR | 1st Qu. | Min. |
| --- | --- | --- | --- | --- | --- | --- | --- | --- | --- |
| Clinical chemistry | 295 | 80 | 4 | 3.2 | 5.3 | 2 | 3 | 1 | 0 |
| Immunoassays incl. serology | 284 | 20 | 3 | 2.4 | 2.4 | 2 | 2 | 1 | 0 |
| Haematology | 290 | 80 | 3 | 2.6 | 5.0 | 2 | 2 | 1 | 0 |
| Coagulation | 278 | 8 | 2 | 1.5 | 1.1 | 1 | 1 | 1 | 0 |
| Urinalysis | 286 | 80 | 2 | 1.9 | 4.8 | 1 | 1 | 1 | 0 |
| Routine molecular diagnostics | 191 | 35 | 3 | 2.8 | 4.5 | 2 | 2 | 1 | 0 |

#### Commercial laboratory

| Specialty | n | Max. | 3rd Qu. | Mean | SD | Median | IQR | 1st Qu. | Min. |
| --- | --- | --- | --- | --- | --- | --- | --- | --- | --- |
| Clinical chemistry | 220 | 90 | 4 | 3.8 | 7.0 | 2 | 3 | 1 | 0 |
| Immunoassays incl. serology | 214 | 28 | 4 | 3.3 | 3.9 | 2 | 3 | 1 | 0 |
| Haematology | 218 | 90 | 3 | 3.3 | 7.0 | 2 | 2 | 1 | 0 |
| Coagulation | 199 | 40 | 2 | 2.0 | 3.3 | 1 | 1 | 1 | 0 |
| Urinalysis | 189 | 70 | 2 | 2.3 | 5.5 | 1 | 1 | 1 | 0 |
| Routine molecular diagnostics | 143 | 70 | 4 | 3.8 | 8.0 | 2 | 3 | 1 | 0 |

## Item 43 - Floor area

### Prompt

Please indicate the **total floor area** of the individual sections in your lab in square meters (for orientation a tennis court has 260 square meters):

- Reception (i.e. accessioning)
- Pre-analytical Workstations (i.e. sorting, centrifuge, aliquots)
- Analytical Workstations
- Post-analytical workstations (sample archiving, result validation)

### Results

#### Combined

| Section | n | Max. | 3rd Qu. | Mean | SD | Median | IQR | 1st Qu. | Min. |
| --- | --- | --- | --- | --- | --- | --- | --- | --- | --- |
| Reception | 920 | 3013 | 50 | 52.1 | 152.6 | 20 | 41 | 9 | 0 |
| Pre-analytical | 920 | 3013 | 50 | 51.4 | 161.5 | 20 | 40 | 10 | 0 |
| Analytical | 920 | 10000 | 200 | 239.6 | 640.9 | 100 | 160 | 40 | 0 |
| Post-analytical | 920 | 3013 | 50 | 56.2 | 151.9 | 20 | 40 | 10 | 0 |

#### Government hospital laboratory

| Section | n | Max. | 3rd Qu. | Mean | SD | Median | IQR | 1st Qu. | Min. |
| --- | --- | --- | --- | --- | --- | --- | --- | --- | --- |
| Reception | 401 | 500 | 40 | 38.0 | 67.1 | 20 | 30 | 10 | 0 |
| Pre-analytical | 401 | 520 | 50 | 38.8 | 58.1 | 20 | 40 | 10 | 0 |
| Analytical | 401 | 10000 | 220 | 226.8 | 615.4 | 100 | 170 | 50 | 0 |
| Post-analytical | 401 | 1000 | 50 | 44.8 | 84.0 | 20 | 40 | 10 | 0 |

#### Private hospital laboratory

| Section | n | Max. | 3rd Qu. | Mean | SD | Median | IQR | 1st Qu. | Min. |
| --- | --- | --- | --- | --- | --- | --- | --- | --- | --- |
| Reception | 296 | 3013 | 30.0 | 45.3 | 185.6 | 15 | 24.0 | 6 | 0 |
| Pre-analytical | 296 | 3013 | 37.2 | 50.6 | 202.0 | 15 | 31.2 | 6 | 0 |
| Analytical | 296 | 9100 | 180.0 | 193.7 | 588.7 | 100 | 140.0 | 40 | 0 |
| Post-analytical | 296 | 3013 | 50.0 | 53.6 | 192.1 | 20 | 41.0 | 9 | 0 |

#### Commercial laboratory

| Section | n | Max. | 3rd Qu. | Mean | SD | Median | IQR | 1st Qu. | Min. |
| --- | --- | --- | --- | --- | --- | --- | --- | --- | --- |
| Reception | 223 | 2000 | 65.0 | 86.5 | 202.4 | 30 | 53.0 | 12 | 0 |
| Pre-analytical | 223 | 2000 | 50.0 | 75.3 | 216.4 | 25 | 40.0 | 10 | 0 |
| Analytical | 223 | 6240 | 210.0 | 323.7 | 739.5 | 100 | 170.0 | 40 | 0 |
| Post-analytical | 223 | 1672 | 76.5 | 80.1 | 181.4 | 25 | 66.5 | 10 | 0 |

## Item 44 - Labor shortage

### Prompt

To what extent do you experience shortage of labor in your laboratory for the roles indicated below (pick one for each of the following)?

- Higher skills
- Lower skills

### Results

#### Combined

|  | None at all | A moderate amount | A great deal |
| --- | --- | --- | --- |
| Higher skills | 217 (24%) | 449 | 254 |
| Lower skills | 379 (41%) | 432 | 109 |

#### Government hospital laboratory

|  | None at all | A moderate amount | A great deal |
| --- | --- | --- | --- |
| Higher skills | 78 (19%) | 195 | 128 |
| Lower skills | 129 (32%) | 205 | 67 |

#### Private hospital laboratory

|  | None at all | A moderate amount | A great deal |
| --- | --- | --- | --- |
| Higher skills | 69 (23%) | 151 | 76 |
| Lower skills | 133 (45%) | 134 | 29 |

#### Commercial laboratory

|  | None at all | A moderate amount | A great deal |
| --- | --- | --- | --- |
| Higher skills | 70 (31%) | 103 | 50 |
| Lower skills | 117 (52%) | 93 | 13 |

# Factor analysis

### Patients per FTE

|  | n | Max. | 3rd Qu. | Mean | SD | Median | IQR | 1st Qu. | Min. |
| --- | --- | --- | --- | --- | --- | --- | --- | --- | --- |
| Combined | 890 | 4500 | 500 | 452 | 606 | 200 | 400 | 100 | 4 |
| Government hospital laboratory | 380 | 4500 | 534 | 483 | 638 | 267 | 409 | 125 | 5 |
| Private hospital laboratory | 293 | 3500 | 500 | 408 | 561 | 200 | 400 | 100 | 4 |
| Commercial laboratory | 217 | 3900 | 500 | 458 | 607 | 200 | 400 | 100 | 8 |

### CFA fit indices

| Fit measure | Value |
| --- | --- |
| RMSEA | 0.06 |
| CFI | 0.91 |
| SRMR | 0.08 |

### Correlation of subscales

|  | OP | ICCP | FS |
| --- | --- | --- | --- |
| OP | 1.00 | 0.68 | 0.71 |
| ICCP | 0.68 | 1.00 | 0.62 |
| FS | 0.71 | 0.62 | 1.00 |
